# Expression analysis suggests that DNMT3L is required for oocyte de novo DNA methylation only in *Muridae* and *Cricetidae* rodents

**DOI:** 10.1186/s13072-023-00518-2

**Published:** 2023-11-04

**Authors:** Lirik Behluli, Alyssa M. Fontanilla, Laura Andessner-Angleitner, Nikolas Tolar, Julia M. Molina, Lenka Gahurova

**Affiliations:** 1 Department of Molecular Biology and Genetics, Faculty of Science, University of South Bohemia, Branisovska 1760, 37005 Ceske Budejovice, Czech Republic; 2 Department of Biological Sciences, Faculty of Sciences and Languages, São Paulo State University “Júlio de Mesquita Filho” - UNESP, Assis, São Paulo Brazil

## Abstract

**Background:**

During early mammalian development, DNA methylation undergoes two waves of reprogramming, enabling transitions between somatic cells, oocyte and embryo. The first wave of de novo DNA methylation establishment occurs in oocytes. Its molecular mechanisms have been studied in mouse, a classical mammalian model. Current model describes DNA methyltransferase 3A (DNMT3A) and its cofactor DNMT3L as two essential factors for oocyte DNA methylation—the ablation of either leads to nearly complete abrogation of DNA methylation. However, *DNMT3L* is not expressed in human oocytes, suggesting that the mechanism uncovered in mouse is not universal across mammals.

**Results:**

We analysed available RNA-seq data sets from oocytes of multiple mammals, including our novel data sets of several rodent species, and revealed that *Dnmt3l* is expressed only in the oocytes of mouse, rat and golden hamster, and at a low level in guinea pigs. We identified a specific promoter sequence recognised by an oocyte transcription factor complex associated with strong *Dnmt3l* activity and demonstrated that it emerged in the rodent clade *Eumuroida*, comprising the families *Muridae* (mice, rats, gerbils) and *Cricetidae* (hamsters). In addition, an evolutionarily novel promoter emerged in the guinea pig, driving weak *Dnmt3l* expression, likely without functional relevance. Therefore, *Dnmt3l* is expressed and consequently plays a role in oocyte de novo DNA methylation only in a small number of rodent species, instead of being an essential pan-mammalian factor. In contrast to somatic cells, where catalytically inactive DNMT3B interacts with DNMT3A, forming a heterotetramer, we did not find evidence for the expression of such inactive *Dnmt3b* isoforms in the oocytes of the tested species.

**Conclusions:**

The analysis of RNA-seq data and genomic sequences revealed that DNMT3L is likely to play a role in oocytes de novo DNA methylation only in mice, rats, gerbils and hamsters. The mechanism governing de novo DNA methylation in the oocytes of most mammalian species, including humans, occurs through a yet unknown mechanism that differs from the current model discovered in mouse.

**Supplementary Information:**

The online version contains supplementary material available at 10.1186/s13072-023-00518-2.

## Introduction

DNA methylation is an epigenetic mark contributing to the determination and maintenance of cell identity through regulation of gene expression patterns and chromatin states. It is substantially reprogrammed during mammalian development, enabling the transition from differentiating somatic cells into germline, and from highly specialised oocyte and sperm into totipotent zygote, followed by the differentiation of individual cell lineages. This reprogramming comprises two waves of nearly complete DNA demethylation followed by de novo DNA methylation establishment [48].

In mammalian oocytes, DNA methylation is established on a largely blank slate during oocyte growth, with the exception of a number of repetitive elements retaining their methylation throughout oogenesis [46]. Current widely accepted model describes DNA methyltransferase 3A (DNMT3A) as the main de novo DNA methylation enzyme in oocytes, catalysing the transfer of the methyl group from S-adenosyl methionine onto unmethylated DNA. DNMT3L acts as an essential co-factor without catalytic activity [5, 25]. Two copies each of DNMT3A and DNMT3L assemble into a heterotetrameric complex [23], and deletion of either of them leads to nearly complete abrogation of oocyte de novo DNA methylation [27, 44, 47]. Despite its oocyte expression, another de novo DNA methyltransferase, DNMT3B, was shown to be dispensable for oocyte de novo DNA methylation [44], while *Muroidea* rodents-specific DNMT3C appears to be restricted to the male germline [3].

Recruitment of the DNMT3A–DNMT3L complex to target genomic regions subject to de novo DNA methylation appears to be exerted by specific post-translational modifications of histone tails [48]. DNMT3A and DNMT3L share the ATRX–DNMT3–DNMT3L (ADD) domain interacting with the N-terminal histone 3 (H3) tail. This interaction is inhibited by H3 lysine 4 di- and tri-methylation (H3K4me2/3) [32, 34, 68]. In contrast to DNMT3A, DNMT3L lacks a large part of the catalytic methyltransferase domain and a Pro–Trp–Trp–Pro motif (PWWP) domain that recognises H3K36me2/3. In vitro studies revealed that the DNMT3A PWWP domain guides DNA methylation to regions with H3K36me3 [14], a mark associated with gene bodies of actively transcribed genes. It was believed that H3K36me3 plays the same DNA methylation guidance role in oocytes, especially considering that the oocyte DNA methylation landscape largely matches the transcribed regions, while methylation is absent from gene bodies of silent genes or from intergenic regions [58]. Moreover, simultaneous depletion of H3K36me2/3 leads to global DNA hypomethylation [65]. However, it was demonstrated that the DNMT3A PWWP domain instead restricts DNA methylation to H3K36me2/3 regions. When the association between DNMT3A and H3K36me2/3 is abolished by D329A substitution in the PWWP domain, oocytes are hypermethylated [26]. Instead, a recent study revealed that amino acid substitutions in the ADD domain of DNMT3A lead to mosaic loss of mouse oocyte DNA methylation. The ADD domain, therefore, likely plays a role in the efficiency and processivity of the DNMT3A–DNMT3L de novo DNA methylation complex [54]. These results suggest that oocyte de novo DNA methylation targeting actively transcribed regions involves additional mechanisms.

Oocyte de novo DNA methylation establishment has been studied in mouse, a classical mammalian model. DNMT3L has been described as an essential factor in oocyte DNA methylation establishment—initially only at the imprinted loci [5], and later, once the development of technologies allowed profiling the whole DNA methylation landscape, at the genome-wide level [27, 44, 47]. Despite *DNMT3L* being transcriptionally silent in human oocytes [36], the DNMT3L-based model described in mouse continues to be accepted as a general mammalian model [8, 11]. Nevertheless, some studies highlight the enigmatic status of DNMT3L due to the lack of expression in human oocytes [13, 42].

Here, we show that the key role of DNMT3L in oocyte DNA methylation establishment is restricted to a specific rodent lineage *Eumuroida* comprising mice, rats, gerbils and hamsters. Its oocyte expression in these species is due to the emergence of an oocyte-specific upstream promoter within the third intron of the upstream gene *Aire*. In other rodent and mammalian lineages, the absence of DNMT3L may have consequences for the interplay between histone modifications and the DNA methylation landscape in oocytes.

## Results

### *Dnmt3l* is transcriptionally silent in the oocytes of rodent naked mole-rat and non-rodent species

**Fig. 1:**
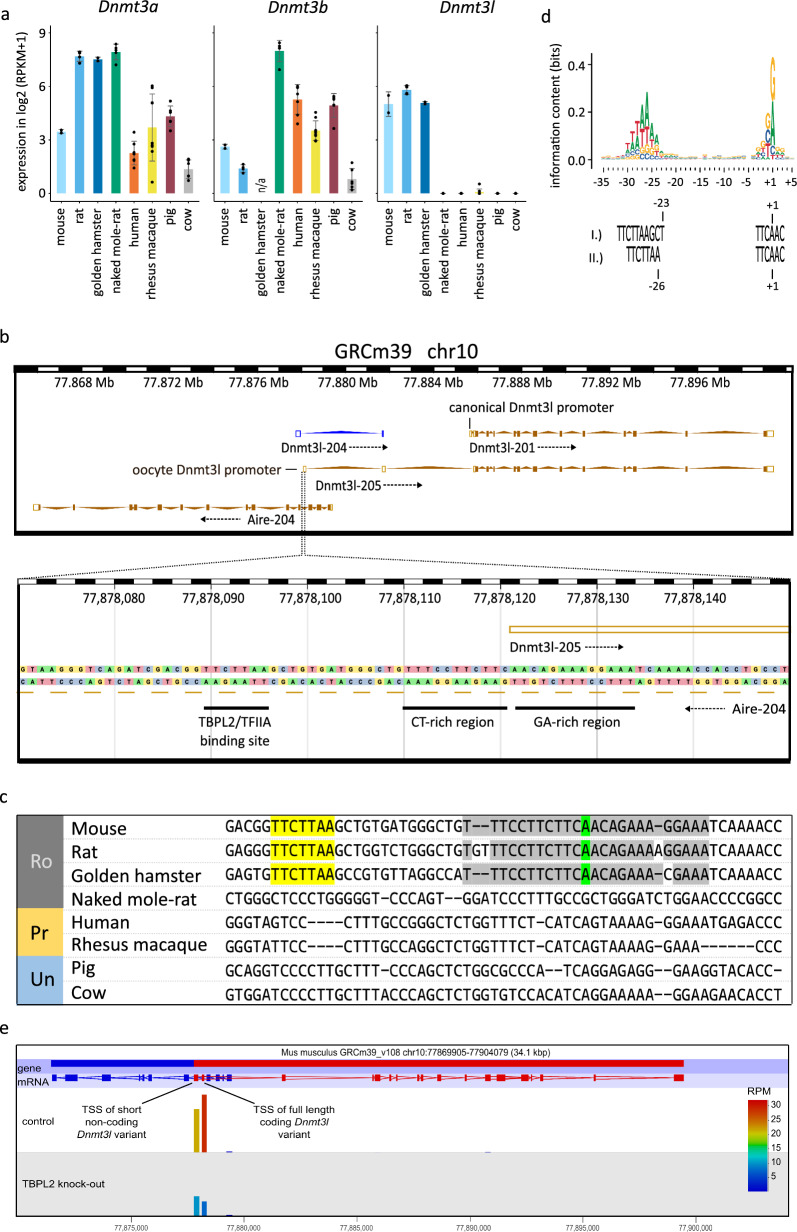
Oocyte *Dnmt3l* expression and promoter sequence. **a** Expression of *Dnmt3* genes across mammalian oocytes using available RNA-seq data sets. RPKM = reads per kilobase of transcript per million reads in the library (in paired-end libraries, fragments instead of reads were quantified). Dots represent individual data points (biological replicates), error bars show standard deviation. **b** Ensembl genome browser screenshot of the mouse *Dnmt3l* gene and its promoter region in single bp resolution. **c** Screenshot of reverse complement of *Aire* intron 3 multiple sequence alignment (only the sequence alignment around TSS and presumable TBPL2/TFIIA binding site is shown) in mammals with available oocyte RNA-seq data sets. The *Dnmt3l* oocyte TSS is highlighted in green, the upstream CT-rich region and downstream GA-rich region surrounding the TSS are highlighted in grey and the presumable TBPL2/TFIIA binding site is highlighted in yellow. Ro rodents, Pr primates, Un ungulates. **d** Visualisation of the TBPL2/TFIIA consensus binding site (adapted from [66] and matching sequences identified in mouse oocyte *Dnmt3l* TSS. Top sequence (I.)) represents the sequence with exactly matching positions upstream of TSS, bottom sequence (II.)) represents best fit for the binding site positioned 3 bp upstream of the exact match. The numbers indicate the position in the mouse sequence respective to the *Dnmt3l* TSS. **e** Screenshot from the program Seqmonk showing SLIC–CAGE read quantification of the first 200 bp from mouse *Dnmt3l* TSSs in control and TBPL2 knock-out oocytes [66]. *RPM* reads per million reads in the library

To analyse oocyte *Dnmt3l* expression beyond mouse and human, we quantified available RNA-seq data of rat, golden hamster, cow, pig, macaque rhesus and our novel data sets of naked mole-rat oocytes. We demonstrated that while *Dnmt3a* and *Dnmt3b* genes are expressed in all species (except golden hamster, where *Dnmt3b* is absent from gene annotation of the MesAur1.0 genome), the expression of *Dnmt3l* is restricted to mouse, rat and golden hamster (Fig. 1a). To confirm that the absence of *Dnmt3l* expression in the naked mole-rat, another rodent species, is not just due to incorrect *Dnmt3l* annotation, we performed a BLAST search of mouse, human and naked mole-rat *Dnmt3l* sequences against the naked mole-rat genome. The existing *Dnmt3l* annotation is the only region that is significantly similar to mouse and human *Dnmt3l*, and no other *Dnmt3l*-like sequence is present in the naked mole-rat genome. Furthermore, we identified that the largest window of sequence identity between naked mole-rat *Dnmt3l* and other *Dnmt3* family genes is 13 bp, confirming that hypothetical reads originating from *Dnmt3l* would not incorrectly map to *Dnmt3a* or *Dnmt3b*.

In mouse, oocyte *Dnmt3l* transcription is initiated from an annotated oocyte-specific promoter localised upstream of the canonical promoter within the third intron of the *Aire* gene encoded on the opposite DNA strand [45] (Fig. 1b). There is an additional isoform of *Dnmt3l* with a transcriptional start site (TSS) within the fourth *Aire* exon and intron; however, it consists of only two exons and lacks a defined coding sequence (ENSMUST00000135316.2 in the Ensembl genome browser). Visual inspection of mouse, rat and golden hamster oocyte RNA-seq data revealed that both isoforms are expressed in mouse and rat oocytes, while only the long protein-coding variant is expressed in golden hamster (although the latter case might be an artefact caused by lower sequencing depth and higher 3’ end bias in golden hamster data). The mouse annotated TSS of the protein-coding oocyte *Dnmt3l* variant is at an adenosine (A) with a CT-rich sequence motif immediately upstream, a GA-rich motif immediately downstream, and a TTCTTAA sequence motif 32 bp upstream of the TSS (Fig. 1b). These sequences are conserved in rat and golden hamster (Fig. 1c) and match a published binding site of TATA box-binding protein-like 2 (TBPL2) assembled into a Transcription factor IIA (TFIIA) complex (Fig. 1d), suggesting that this complex is likely to drive oocyte *Dnmt3l* expression. The TBPL2/TFIIA complex has been previously shown to regulate transcription from oocyte-specific TSSs because of its specific TSS recognition code [66]. Quantification of *Dnmt3l* expression and its oocyte TSS activity using published RNA-seq and super-low input carrier–CAGE (SLIC–CAGE) data sets [66], respectively, revealed that TBPL2 knock-out leads to significant downregulation of mouse oocyte *Dnmt3l* (Fig. 1e and Additional file 1: Fig. S1a for SLIC–CAGE results, while the results of RNA-seq can be found in [66]). It should be noted that SLIC–CAGE data confirm that both full length oocyte *Dnmt3l* and short noncoding transcript are expressed in mouse oocytes, but only the TSS of full-length *Dnmt3l* appears to be substantially regulated by the TBPL2/TFIIA complex, as it is statistically significantly downregulated in the TBPL2 knock-out SLIC–CAGE data set. Although the *TBPL2* gene and its oocyte expression are conserved in all tested mammalian species with oocyte RNA-seq data sets, the *Dnmt3l* oocyte promoter sequence motif is not conserved in species, where *Dnmt3l* is transcriptionally silent in oocytes (Fig. 1c).

To exclude the possibility of Dnmt3l expression from the identified promoter in other mammalian lineages, we analysed sequences of *Aire* intron equivalent to mouse intron 3 of additional mammalian species (10 primates, 10 Laurasiatheria species, 2 Afrotheria species and 1 Xenarthra species) with genome sequences available in the Ensembl genome database and with an annotated *Aire* gene (Additional file 6: Table S1), and confirmed the absence of TTCTTAA or a similar promoter sequence motif, as well as the TSS sequence. These results associate the evolutionary emergence of a sequence recognised by an oocyte TF complex with *Dnmt3l* expression in the oocytes, employing the oocyte-specific promoter.

### Oocyte *Dnmt3l* expression is restricted to* Muridae* and* Cricetidae* rodents

**Fig. 2:**
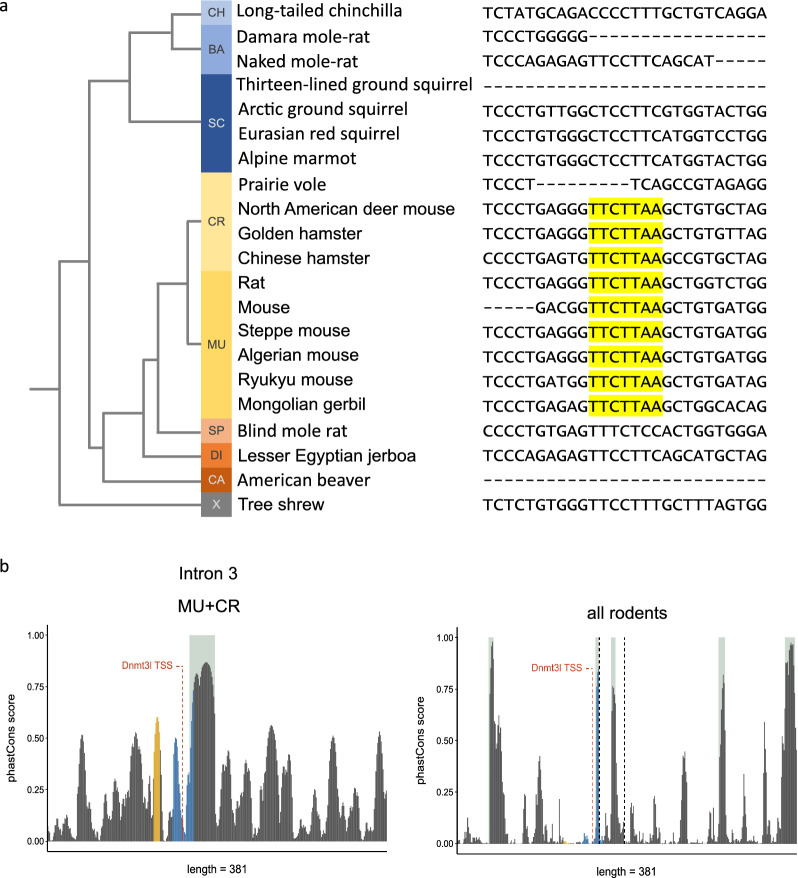
*Dnmt3l* oocyte promoter sequence conservation in rodents. **a** Screenshot of a reverse complement of *Aire* intron 3 multiple sequence alignment with highlighted conserved promoter sequences in rodents with available sequences in Ensembl genome database. CH *Chinchillidae*, BA *Bathyergidae*, SC *Sciuridae*, CR *Cricetidae*, MU *Muridae*, SP *Spalacidae*, DI *Dipodida*e, CA *Castoridae*, X *Scandentia*. **b** PhastCons scores across mouse *Aire* intron 3 in *Muridae* and *Cricetidae* (except prairie vole) rodents on the left and all rodents with available sequence in the Ensembl genome database on the right. Regions highlighted in light grey indicate highly conserved regions identified by PhastCons. The presumed TBPL2/TFIIA binding motif is highlighted in yellow and CT-rich and GA-rich regions surrounding the TSS are highlighted in blue

To obtain more insights into *Dnmt3l* expression across rodents, we downloaded *Aire* intron sequences of all rodents available in the Ensembl genome database and of a species of tree shrew, which belongs to *Scandentia*, a sister clade to glires comprising rodents and lagomorphs (Additional file 6: Table S2). Based on the manual analysis of exon and intron sequences, we identified an intron corresponding to mouse intron 3 in each species except kangaroo rat. Moreover, we excluded guinea pig from the analysis, as the sequence of the corresponding intron is not determined at the nucleotide level (unknown nucleotides in the sequence). A multiple sequence alignment (Additional file 5) of these intron sequences revealed that the TTCTTAA promoter sequence motif is present only in the *Eumuroida* rodent clade, comprising families *Muridae* (mice, rats, gerbils) and *Cricetidae* (hamsters), with the exception of the prairie vole, where a larger region surrounding the promoter sequence was deleted (Fig. 2a). The TSS, including upstream CT-rich and downstream GA-rich motif, is also conserved in the prairie vole (Additional file 1: Fig. S1b). In the *Spalacidae* family (exemplified by blind mole rat), a sister group to *Eumuroida* rodents within superfamily *Muroidea*, as well as in the family *Sciuridae* (squirrels), predicted TBPL2/TFIIA binding motif is conserved to some extent, although with multiple mismatches. Region surrounding potential TSS is conserved only partially. Moreover, in blind mole rat, there is a relatively large insertion (> 100 bp) between predicted binding site and region homologous to TSS. These sequences are probably not compatible with *Dnmt3l* oocyte transcription. For example, a similar sequence homologous to predicted TBPL2/TFIIA binding motif in naked mole-rat does not drive oocyte *Dnmt3l* expression. Nevertheless, this suggests that the ancestral sequence in rodents or a subgroup of rodents was favourable for the emergence of sequence motif recognised by an oocyte TF complex, driving oocyte *Dnmt3l* transcription.

To confirm that the oocyte *Dnmt3l* promoter region (particularly the presumable TBPL2/TFIIA binding motif and CT-rich and GA-rich regions upstream and downstream of the TSS, respectively) became evolutionarily conserved after its emergence, we analysed local conservation scores across the first five *Aire* introns in rodents using PhastCons [37]. We demonstrated that the presumable TBPL2/TFIIA binding motif forms a relatively conserved peak in *Muridae* and *Cricetidae* rodents with oocyte *Dnmt3l* expression, while there is no conservation of this region if all rodents are considered. The conservation is similar for the CT-rich region upstream of *Dnmt3l* oocyte TSS. In contrast, the GA-rich sequence downstream of the TSS is universally conserved across rodents (Fig. 2b). However, intron 3 and other *Aire* introns appear to contain other regions with similar or higher conservation scores, suggesting that the *Dnmt3l* promoter region is not unique in its high conservation (Additional file 2: Fig. S2).

### Evolutionarily novel oocyte *Dnmt3l* promoter in guinea pigs drives weak oocyte expression

**Fig. 3:**
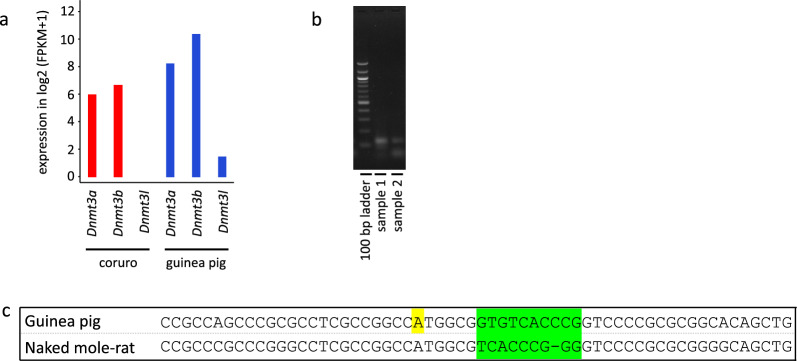
*Dnmt3l* oocyte expression in guinea pig and coruro. **a** Expression of *Dnmt3* genes in coruro and guinea pig oocytes (single replicates). FPKM = fragments per kilobase of transcript per million fragments in the library. **b** PCR with reverse transcribed RNA from guinea pig oocytes amplifying region of exons 8–10 of the annotated *Dnmt3l* gene **c** Sequence alignment screenshot of a region surrounding Aire TSS in guinea pig and naked mole rat. The guinea pig *Dnmt3l* TSS is highlighted in yellow, the low conservation region in the otherwise highly conserved sequence is highlighted in green

To further confirm the lack of oocyte *Dnmt3l* expression in rodents outside the *Muridae* and *Cricetidae* families, we performed RNA-seq on oocytes of guinea pig and coruro. Both species belong to the *Caviomorpha* lineage of *Hystricognathi* rodents, while naked mole-rat belongs to the *Phiomorpha* lineage. Guinea pig is representative of the *Caviidae* family within the *Cavioidea* superfamily, while coruro, closely related to degu, is representative of the *Octodontidae* family within the *Octodontoidea* superfamily. Because coruro genome assembly is not available, we mapped its data to degu *Dnmt3a*, *Dnmt3b* and *Dnmt3l* sequences with less stringent mapping parameters. All three genes appear to be under similar evolutionary pressures based on dN/dS ratios according to the best fitting model for each gene (Additional file 6: Table S3) [1], confirming that coruro reads should map to degu sequences with comparable probabilities. We showed relatively high expression of coruro *Dnmt3a* and *Dnmt3b*, while no reads were mapped to *Dnmt3l* (Fig. 3a). In contrast, mapping of guinea pig reads revealed very weak oocyte *Dnmt3l* expression (Fig. 3a) that was confirmed by qPCR using primers specific for *Dnmt3l* mRNA comprising exons 8–10 (Fig. 3b) and sequence verification of the PCR product.

According to the Ensembl annotation for the guinea pig Cavpor3.0 genome, the *Dnmt3l* TSS is located closely upstream of the *Aire* TSS encoded on the opposite DNA strand. This appears unique across rodents and other mammals, as the canonical (embryonic) *Dnmt3l* TSS is located much more upstream of the *Aire* TSS in other species, while the TSS of the oocyte isoform is within the third *Aire* intron. As we mentioned previously, a large part of the corresponding guinea pig *Aire* intron is not deciphered at the nucleotide level. We wondered whether the oocyte *Dnmt3l* transcript could potentially start within this intron; however, sequencing of this region revealed that the oocyte promoter sequence is not conserved in guinea pig (Additional file 3: Fig. S3). To identify what could be driving weak oocyte *Dnmt3l* expression in guinea pig, we compared sequences ± 250 bp around annotated *Aire* TSS between guinea pig and naked mole-rat. The whole sequence was largely conserved, including the immediate region around the annotated *Dnmt3l* TSS in guinea pig, with the exception of a 9 bp region located 6 bp downstream, suggesting that this sequence could potentially regulate *Dnmt3l* expression in guinea pig (Fig. 3c). To confirm that this evolutionarily novel *Dnmt3l* promoter drives weak transcription in oocytes, we manually inspected RNA-seq and found out that no read maps to the annotated first exon. Moreover, we designed two primer pairs with forward primers specific for exon 1, but neither reaction produced specific products (data not shown). Therefore, we cannot confirm that weak guinea pig oocyte *Dnmt3l* expression initiates at the annotated TSS.

### DNMT3L does not appear to play a substantial role in retrotransposon silencing

Due to the restricted rodent lineage specificity of both the *Dnmt3c* gene and oocyte *Dnmt3l* isoform, it is tempting to speculate about the similar functional role of proteins encoded by these genes in male and female germline, respectively. The *Muroidea* rodents-specific *Dnmt3c* gene plays a role in suppressing evolutionary young retrotransposons in the male germline and shows convergence with elements targeted by the piRNA pathway [3]. This gene is evolutionarily older than the oocyte *Dnmt3l* isoform, as it was also detected in the *Spalacidae* family [3], while we show that the oocyte *Dnmt3l* TSS appeared after splitting of *Muroidea* rodents into *Spalacidae* and *Eumuroida* lineages.

**Fig. 4:**
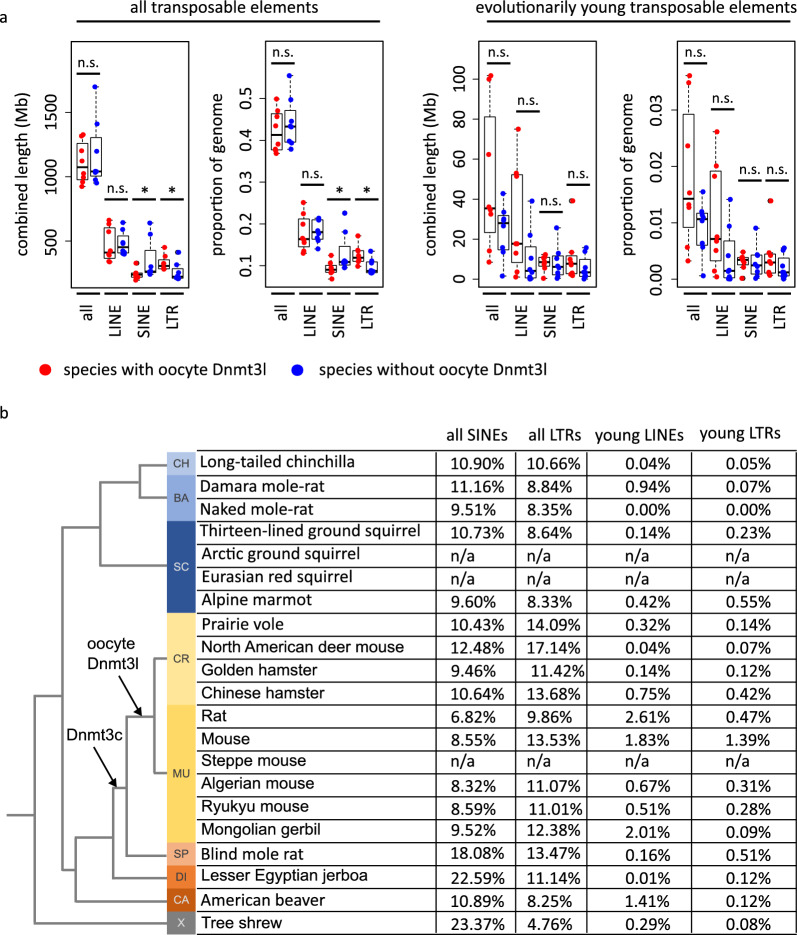
Oocyte *Dnmt3l* does not play a substantial role in retrotransposon silencing. **a** Combined length of and proportion of genome represented by all transposons and selected retrotransposon classes for all transposons and evolutionary young transposons in rodent species with and without oocyte *Dnmt3l*. **b** Proportion of the genome represented by all SINEs, all LTRs, evolutionarily young LINEs and LTRs in individual species with and without oocyte *Dnmt3l*

To obtain more insights into the potential association between retrotransposon activity and the emergence of oocyte *Dnmt3l* expression, we first compared the genomic content of all retrotransposons and evolutionarily young (and, therefore, still potentially active, or recently active) retrotransposons as estimated by Osmanski et al. [33] in rodents with and without predicted oocyte *Dnmt3l* expression. We detected significantly fewer SINE elements and significantly more LTR elements in species with oocyte *Dnmt3l*. We also noticed that species with oocyte *Dnmt3l* tend to have more evolutionarily young retrotransposons, especially of LINE and LTR classes (although this difference is not statistically significant) (Fig. 4a). However, there is no clear trend visible when visualised in the phylogenetic context (Fig. 4b). On the other hand, no such trend is associated with the emergence of *Dnmt3c* either (Fig. 4b).

In addition, we analysed a published RNA-seq data set from *Dnmt3l* knock-out (*Dnmt3l*-ko) fully grown mouse oocytes [44] to determine whether abrogation of DNMT3L-dependent DNA methylation leads to transcriptional reactivation of specific retrotransposons compared to control oocytes. No retrotransposon class was upregulated in *Dnmt3l*-ko oocytes (Additional file 4: Fig. S4a). The analysis of individual TE subfamilies using TEtranscripts [24] identified eight that are differentially expressed with probability 0.95 or higher (Additional file 4: Fig. S4b). However, seven of these are downregulated in *Dnmt3l*-ko oocytes. More detailed analysis of the single upregulated subfamily, ERVB4_3-LTR_MM, revealed that most of the reads originate in a single insertion that is at the 3’ end of a longer unannotated transcript that is strongly upregulated in *Dnmt3l*-ko oocytes (data not shown). Therefore, we concluded that no TE subfamily appears to be derepressed if DNMT3L-dependent DNA methylation is abrogated. Retrotransposons with the highest activation in *Dnmt3c*-ko testes (L1Md_A, L1Md_T, L1Md_Gf, L1_Mm, MMERVK10C-int) [3] are silenced in both control and *Dnmt3l*-ko oocytes (data not shown). As mouse has one of the highest proportions of evolutionarily young LINE and LTR retrotransposons [33], we compared the expression of the most abundant subfamilies of these classes (LINE-L1, LTR–ERVK, LTR–ERVL, LTR–MaLR) and demonstrated that neither retrotransposon is substantially upregulated in *Dnmt3l*-ko oocytes. A relatively small number of specific insertions of various retrotransposon classes appear to be upregulated in *Dnmt3l*-ko oocytes; however, an equal or larger number of insertions of the same element is downregulated in *Dnmt3l*-ko oocytes (Additional file 1: Fig. S4c). Therefore, unlike *Dnmt3c,* which has a demonstrated role in silencing evolutionarily young retrotransposons in mouse testes, *Dnmt3l* does not appear to play a substantial role in retrotransposon silencing in mouse oocytes.

### Absence of DNMT3L is not compensated by the expression of inactive DNMT3B isoforms in the oocytes of mammals outside Eumuroida rodents

**Fig. 5:**
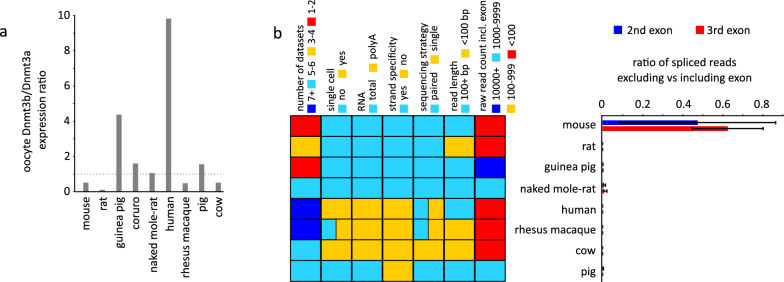
Inactive DNMT3B isoform is not likely to replace DNMT3L. **a** *Dnmt3b/Dnmt3a* oocyte expression ratio across mammalian species; the dotted line marks a ratio of 1. **b** Right: ratio of spliced reads that splice out the two exons that are spliced out in mouse inactive *Dnmt3b* isoforms to spliced reads that include the two exons. Exons are marked in their order from the 3’ end of the transcript. Left: technical information about data sets used for the analysis, from left to right—number of data sets analysed, whether the data sets are from single oocytes, whether total or polyA-selected RNA was used, whether the generated library is strand-specific, whether the library was sequenced single-end or paired-end, sequencing read length and average raw read count for spliced reads that include the two analysed exons. Two colours indicate different parameters if multiple sources of data sets were used (human and rhesus macaque)

It was previously suggested that DNMT3L may be substituted by an inactive isoform of DNMT3B in human oocytes [13], as was observed in somatic cells [15, 67]. This is supported by generally higher expression levels of *Dnmt3b* in species without oocyte *Dnmt3l *(Figs. 1a, 3a) and a higher *Dnmt3b* to *Dnmt3a* expression ratio (> 1 in all except cow and rhesus macaque) (Fig. 5a). However, our analysis of the RNA-seq data sets did not detect a correlation between the absence of oocyte *Dnmt3l* and increased expression of *Dnmt3b* isoforms homologous to the mouse inactive isoforms *Dnmt3b3*, *Dnmt3b4* and *Dnmt3b7* (Fig. 5b), which are characterised by second and third exons from the 3’ end being spliced out. Instead, such isoforms were detected at substantial expression levels only in mouse oocytes. However, this result could be affected by the technical differences across libraries from different species (Fig. 5b). Visual inspection of spliced reads across *Dnmt3b* in individual species did not reveal the presence of novel potentially inactive *Dnmt3b* isoforms. Therefore, it remains to be elucidated what substitutes DNMT3L in a tetramer with DNMT3A, and whether both DNMT3A and DNMT3B are essential for oocyte de novo DNA methylation in species without oocyte DNMT3L.

## Discussion

Here, we challenge the current model that DNMT3L is generally required for de novo DNA methylation in mammalian oocytes. Instead, we show that its expression in oocytes is driven by an oocyte-specific upstream promoter that emerged in the *Eumuroida* rodent lineage, restricting oocyte *Dnmt3l* activity to mice, rats, gerbils and hamsters. Our study demonstrates that results obtained by studying mouse as a model cannot be easily extrapolated to all mammals, as was also shown previously in other biological contexts [7, 19, 21, 22, 35, 43, 51, 56, 57], or even to all rodents. Moreover, it highlights the importance of examining non-model species to both elucidate the universality of mechanisms first identified in mouse and explore the variability across mammals.

As we did not detect the expression of inactive *Dnmt3b* isoforms in the oocytes of species outside *Eumuroida* rodents, we hypothesise that DNMT3A may act in a complex with active DNMT3B, or potentially form a homotetramer [16]. This interaction was previously shown in P19 somatic cells in the absence of DNMT3L; however, inactive DNMT3B was also found in the complex [28]. Long read sequencing of mammalian oocyte transcriptomes could shed more light on which *Dnmt3b* isoforms are expressed and, therefore, have the potential to form a complex with DNMT3A. To date, only short read Illumina data sets are available for all species except mouse. When we attempted to analyse if any annotated exons are spliced out using the same approach, as shown in Fig. 5b, no exon in any species except mouse showed notable proportion of reads splicing the exon out compared to reads including the exon in the transcript (data not shown). This suggests that full length *Dnmt3b* isoforms are predominantly expressed irrespective of the presence or absence of oocyte DNMT3L.

Although it was originally believed that the DNMT3A–DNMT3L complex was targeted to the gene bodies of active genes in oocytes through DNMT3A PWWP domain-mediated recognition of H3K36me3 [48], recent findings showed that abolition of the association between DNMT3A and H3K36me2/3 in mouse oocytes does not result in hypomethylation of active gene bodies [26]. Instead, such oocytes are hypermethylated, particularly at transcriptionally inactive regions. Therefore, we hypothesise that the ADD domains of DNMT3L and/or DNMT3A target de novo DNA methyltransferase complex activity to regions lacking methylation at H3K4 (as was recently shown for DNMT3A [54]), while the DNMT3A PWWP domain further refines the preference by association with H3K36me2/3. Nevertheless, correct genomic landscapes of both H3K4 and H3K36 methylation are required for appropriate DNA methylation establishment [10, 19–21, 49, 65]. DNMT3B, including its inactive isoforms, contains the PWWP domain recognising H3K36me2/3, while this domain is absent from DNMT3L. Additional PWWP domains in the DNMT3A–DNMT3B complex compared to DNMT3A–DNMT3L complex may, therefore, affect the recognition of the histone modification landscape permissive of de novo DNA methylation by enhancing the role of H3K36me2/3 in complex targeting. This might have consequences for the overall oocyte epigenomic landscape and could explain the differences in profiles of individual histone modifications between species with and without oocyte DNMT3L. In particular, large non-canonical H3K4me3 domains in mouse and rat oocytes might serve to exclude de novo DNA methylation targeted primarily by ADD domains of DNMT3A–DNMT3L complex. In contrast, these domains appear to be absent in human oocytes and less prominent in cow and pig oocytes [29], where de novo DNA methylation might be targeted primarily by H3K36me2/3 recognised by PWWP domains of the DNMT3A–DNMT3B complex.

In mouse, *Dnmt3l* is transcriptionally silent in primordial germ cells and its expression starts during oocyte growth [18]. Limitation of this study is that we cannot exclude the possibility that in species without oocyte *Dnmt3l* expression, *Dnmt3l* is expressed in primordial germ cells and then persists as a protein until oocyte growth when DNA methylation is established. Nevertheless, published proteomes of human and bovine oocytes do not detect DNMT3L protein [12, 31], while DNMT3B is detected. In mouse oocytes, DNMT3L protein was detected [59]. Moreover, *Dnmt3l *expression could occur earlier in the oogenesis and be no longer detectable at mRNA or protein level in fully grown oocytes while still playing a role in de novo DNA methylation establishment. Therefore, the role of DNMT3L in mammals outside *Eumuroida* rodents cannot be conclusively rejected or confirmed without generation of female germ line-specific DNMT3L-knock out individuals.

In mouse, it was shown that DNMT3L is essential for DNA methylation at transposable elements crucial for correct development of male germ cells [4]. *Dnmt3l* is transcribed from different promoters in male and female germline in mouse [45], suggesting independent regulation of expression. Therefore, the restriction of oocyte *Dnmt3l* expression to *Eumuroida* rodents cannot be extrapolated to male germline. Moreover, DNMT3L was shown to play a role in DNA methylation of extra-embryonic ectoderm [2]. Although the requirement for DNMT3L in DNA methylation in male germline or placenta was not yet studied in any mammalian species outside mouse, it may be conserved, explaining the evolutionary stable presence of *Dnmt3l* gene in therian mammals.

We are aware that our conclusions are primarily supported by the identification of sequence motifs associated with *Dnmt3l* transcription in mouse and their absence across mammalian species outside *Eumuroida* rodents. The regulation of oocyte *Dnmt3l* TSS by TBPL2/TFIIA complex binding to the identified motif should be confirmed, for example, by TBPL2 chromatin immunoprecipitation. We do not exclude the possibility that *Dnmt3l* expression in other species could be regulated by different sequence motifs recognised by different oocyte TFs, or even evolutionarily more distant TBPL2/TFIIA complex—for example, in squirrels with relatively high similarity to mouse sequence motifs. Nevertheless, the analysis of available mammalian oocyte RNA-seq data sets strongly supports our conclusion that DNMT3L is not a universal mammalian de novo DNA methylation factor, as we could not detect oocyte *DNMT3L* expression in the oocytes of human, macaque rhesus, cow, pig and even another rodents, naked mole-rat and coruro, and only very weakly in guinea pig. Due to the very low expression level, it is unlikely that DNMT3L in guinea pig forms a tetramer complex with DNMT3A and plays an essential role in oocyte de novo DNA methylation establishment.

The development of low input and single cell approaches for profiling transcriptomes and epigenomes, together with high conservation of histone sequences and a high number of mammalian species with available genome sequences, facilitates interrogation of transcription, DNA methylation and histone modification landscapes in the oocytes of non-model species even when only scarce samples are available. This was already proven by mapping transcriptomes and epigenomes of the oocytes and early embryos of not only mouse and human, but also emerging mammalian models, such as cow and pig, revealing differences in oocyte epigenomes and different patterns of epigenome reprogramming after fertilisation [29]. Applying these approaches to non-model mammalian species would thus shed more light on the mechanisms of oocyte de novo DNA methylation establishment and its interplay with the histone modifications landscape, as well as the mechanisms of oocyte-to-zygotic transition at the transcriptome and epigenome levels. Importantly, it will also contribute to the understanding of the variability of these processes across mammals, reflecting different evolutionary histories and having functional consequences for the reproductive biology of individual species.

## Conclusion

Our results suggest that the *Dnmt3l* oocyte-specific promoter emerged in the *Eumuroida* rodent lineage. Therefore, *Dnmt3l* is likely to be expressed and consequently to play a role as an essential de novo DNA methylation factor only in the oocytes of these species. In contrast to *Dnmt3c,* which is required for silencing-specific evolutionarily young retrotransposons, *Dnmt3l* has a genome-wide role without a substantial effect on retrotransposon silencing. The mechanism governing de novo DNA methylation in the oocytes of most mammalian species including humans remains to be elucidated, as our results do not support the hypothesis that inactive isoforms of DNMT3B substitute DNMT3L in tetramers with DNMT3A.

## Methods

### Oocyte collection

Experimental procedures were carried out in accordance with EU directive 2010/63/EU (for animal experiments). Ovaries were dissected from 5 freshly sacrificed naked mole-rat females 1–8 years old, 3 guinea pig females 6–9 months old and one coruro female 2 years old. Oocytes (fully grown and at the late stage of growth) were isolated from ovaries by mechanical disruption of follicles in M2 medium (pre-warmed at 37 °C for a minimum of 2 h), and cumulus cells were removed by repeated mouth pipetting with a narrow glass pipette. Clean oocytes were washed through several 1X PBS drops.

RNA extraction and RNA-seq library preparation.

Immediately after oocyte collection, total RNA was extracted using an ARCTURUS PicoPure RNA Isolation Kit (Thermo Fisher Scientific; Cat. No. KIT0204) following the manufacturer’s protocol. Due to the number of isolated oocytes, naked mole-rat samples were processed individually for each female, while guinea pig oocytes were merged together from all three females. Ribosomal RNA was depleted with NEBNext rRNA Depletion Kit v2 (Human/Mouse/Rat) with sample purification beads (NEB, E7405) according to the manufacturer’s instructions, including DNase treatment by TURBO DNase (Thermo Fisher Scientific, AM2238) followed by library preparation using NEBNext Ultra II Directional RNA Library Prep with sample purification beads (NEB, E7765S) according to the manufacturer’s instructions. The library was sequenced as 150 bp paired-end reads on an Illumina NovaSeq 6000 SP.

### Data processing and mapping

In addition to our newly generated RNA-seq data set from naked mole-rat, guinea pig and coruro oocytes, we downloaded and processed additional publicly available data sets from oocytes of multiple mammalian species: mouse (GSE70116, GO2 and FGO only) [58], rat (GSE112622) [6], golden hamster (GSE86470) [17], cow (GSE61717) [41], pig (GSE108900) [53], rhesus macaque (GSE103313, GSE86938) [9, 60] and human (GSE36552, GSE101571) [62, 63]. In addition, we downloaded and processed SLIC–CAGE data sets from control and TBPL2 knock-out oocytes (E-MTAB-8866) [66] and RNA-seq data sets from control and *Dnmt3l*-ko oocytes (DRA000570). Publicly available data sets were trimmed with Trim Galore! v0.4.1 or Trim Galore! V0.6.2 (E-MTAB-8866, DRA000570) with default parameters, specifying whether the reads were generated in single end or paired end mode. Naked mole-rat data were trimmed with Trim Galore! v0.6.2 in paired end mode. Trimmed RNA-seq data were mapped with Hisat2 v2.0.5 to respective genomes: GRCm38 and GRCm39 for mouse, Rnor_6.0 for rat, MesAur1.0 for golden hamster, Cavpor3.0 for guinea pig, HetGla_female_1.0 for naked mole-rat, Mmul_8 for rhesus macaque, GRCh38 for human, UMD3.1 for cow and Sscrofa11.1 for pig. SLIC–CAGE data sets (E-MTAB-8866) were mapped with Bowtie2 v2.5.1. Coruro data were mapped to degu Dnmt3a (XM_023711114.1), Dnmt3b (XM_004630806.2) and Dnmt3l (XM_023716073.1) sequences downloaded from the NCBI Nucleotide database with Bowtie2 v2.5.1 specifying the local alignment option. Quantified RNA-seq data sets from control and TBPL2 knock-out oocytes were analysed as published [66]. When analysing TE expression in mouse oocytes at the level of whole families, reads were mapped to artificially merged repeat sequences for the GRCm39 mouse genome downloaded from the UCSC genome browser separated by the NNNNN sequence using Bowtie2 v2.5.1.

### Data analysis

The expression of DNMTs was visually inspected and quantified in SeqMonk v1.47.2 (https://www.bioinformatics.babraham.ac.uk/projects/seqmonk/). To confirm that *Dnmt3l* is annotated at the correct position in the naked mole-rat genome, *Dnmt3l* sequences from the NCBI database for mouse, human and naked mole-rat were blasted to the naked mole-rat HetGla_female_1.0 genome using NCBI Genome Workbench. A custom Python script was prepared to identify the length of regions with continuous sequence identity between *Dnmt3l *vs *Dnmt3a* and *Dnmt3b*. Expression levels were log_2_(RPKM + 1)-transformed in R and plotted using ggplot2 [61].

Aire intron sequences for the genome versions listed above and in Additional file 6: Tables S1 and S2 were downloaded from the Ensembl genome database and were converted to their reverse complements. Multiple sequence alignments of the original sequences and reverse complements were performed using MUltiple Sequence Comparison by Log-Expectation (MUSCLE) tool [30], with ClustalW output format and default parameters. The alignment and individual sequences of species without identified homologous sequence for predicted TBPL2/TFIIA binding site were further manually checked for the presence for such sequence according to the sequence similarity and sequence similarity of the surrounding sequences. PhastCons analyses were performed using the PhastWeb interface [37], with expected length = 7 and default target coverage and rho values. Multiple sequence alignments and Newick trees generated by ClustalW were used as input files, and the mouse sequence was consistently used as a reference sequence. PhastCons scores were visualised using ggplot2 [61].

dN/dS ratios were computed for each of the *Dnmt3* genes using the CODEML software of PAML v4.9j [64] by running a null model (M0) that assumes equal dN/dS ratios across all branches of the phylogeny, and two branch models that assume differences in dN/dS ratios between foreground and background branches. For the first branch model *Hystricognathi* were designated as foreground branches, whereas only *Fukomys damarensis* and *Heterocephalus glaber* were set as foreground branches for the second. Chi-squared tests were conducted at 1% and 5% significance levels using the LRT statistics from each model [1]. The phylogenies and multiple sequence alignments used as CODEML input were generated using phytools [40] and MACSE v2.07 [38, 39], respectively. Rooted and unrooted consensus trees were created based on a subset of 1,000 node-dated phylogenetic trees downloaded from VertLife [55]. Sequences included in the alignments (listed in Additional file 6: Table S4) were downloaded from the NCBI Nucleotide database. For the alignment runs, internal frameshifts were replaced with “---”, while internal stop codons were replaced with “NNN”. The alignments were also trimmed, so that they start and end with at least 80% of the sequences having a nucleotide.

Expression of TE subfamilies (raw counts) was quantified using TEtranscripts v2.2.3 [24], using GRCm39 mouse genome and corresponding TE annotation available on the TEtranscripts website (https://labshare.cshl.edu/shares/mhammelllab/www-data/TEtranscripts/TE_GTF/). This was followed by the identification of differentially expressed TEs using NOISeq-sim within NOISeq [52]. For quantification of individual TE insertion expression, only elements with > 50 insertions outside gene annotation with > 5 reads (mapped using Hisat2 v2.0.5 reporting random one position for multimapping reads) in either condition were considered. TE annotation was obtained from UCSC genome browser for GRCm38.

### DNA extraction, PCR and sanger sequencing

To obtain the missing guinea pig *Aire* intron 3 sequence, DNA was extracted from guinea pig liver using ethanol precipitation. PCR on the region of interest was performed using Phusion High-Fidelity PCR Master Mix with HF buffer (Thermo Fisher Scientific, F531S) and primers with sequences: forward AAGACTCCTGTACTGCCACC and reverse GCTGCAACTCCGAATTACCC. A single PCR product was confirmed by gel electrophoresis, purified by a Monarch PCR & DNA Cleanup Kit (NEB, T1030L) and sequenced by Sanger sequencing.

To confirm guinea pig *Dnmt3l* oocyte expression, RNA extracted from oocytes was treated with TURBO DNase (Thermo Fisher Scientific, AM2238), reverse transcribed with SuperScript III Reverse Transcriptase (Thermo Fisher Scientific, 18080093) and amplified by PCR using AMPIGENE Taq Mix (Enzo, ENZ-NUC100-0200) with primers-specific exons 8 + 10 (forward AGAGCTGATGAGTTTGGGCT, reverse GCCGTACACCAGGTCAAATG) of the annotated guinea pig *Dnmt3l* transcript ENSCPOT00000004115.3. PCR product was purified by a Monarch PCR & DNA Cleanup Kit (NEB, T1030L) and sequenced by Sanger sequencing.

### Supplementary Information

**Additional file 1: Figure S1.** *Dnmt3l* regulation by the TBPL2/TFIIA complex and its TSS conservation across rodents. (a) Mouse oocyte SLIC–CAGE read quantification of the first 200 bp of mRNAs in control and TBPL2 knock-out oocytes [66]. The TSS of full-length oocyte *Dnmt3l* is highlighted in red. (b) Multiple sequence alignment of the reverse complements of *Aire* intron 3 sequences in rodents with available sequences in the Ensembl genome database. TSS and surrounding CT-rich and GA-rich sequences are highlighted in species with known or predicted *Dnmt3l* oocyte expression. Asterisk (*) and dollar ($) mark positions in blind mole rat and lesser Egyptian jerboa with insertions (without homologous sequences in other species) that are not visualised. Unser the alignment, the lengths of the insertions are indicated. The simplified phylogeny (in light grey) to the left of the species names is based on [50].

**Additional file 2: Figure S2.** PhastCons results for Aire introns 1–5. PhastCons scores across mouse *Aire* introns 1–5 in *Muridae* and *Cricetidae* (except prairie vole) rodents. In intron 3, the region highlighted in light grey represents the highly conserved region identified by PhastCons. The promoter sequence is highlighted in yellow and CT-rich and GA-rich regions surrounding the TSS are highlighted in blue.

**Additional file 3: Figure S3.** Oocyte *Dnmt3l* promoter within *Aire* intron 3 is not conserved in guinea pig. (a) Multiple sequence alignment of the *Aire* intron 3 sequence, including the newly sequenced guinea pig sequence. The presumed TBPL2/TFIIA binding motif is highlighted in yellow. GP guinea pig, NMR naked mole-rat.

**Additional file 4: Figure S4.** Expression changes of retrotransposon insertions in *Dnmt3l*-ko mouse oocytes. (a) Percentage of reads mapped to retrotransposon classes in *Dnmt3l-*ko mouse oocytes compared to the control. (b) TE subfamilies that were identified as differentially changing in *Dnmt3l*-ko (ko) oocytes compared to wild type (wt). (c) Proportions of insertions that are > twofold upregulated (up), > twofold downregulated (down) or with smaller change in expression (same) in *Dnmt3l*-ko mouse oocytes compared to the control.

**Additional file 5. **Multiple sequence alignment of rodent *Aire* intron 3 sequences.

**Additional file 6: Table S1.** List of additional mammalian species with genome sequences in the Ensembl database and with annotated Aire gene that were used for analysis for the presence of a potential *Dnmt3l* oocyte promoter within the *Aire* intron equivalent to mouse intron 3. **Table S2.** List of all rodents with genome sequences in the Ensembl database and with annotated *Aire* gene and a tree shrew that were used for analysis for the presence of a potential *Dnmt3l* oocyte promoter within the *Aire* intron equivalent to mouse intron 3. **Table S3.** Results of dN/dS ratio analysis for *Dnmt3* genes in rodents. **Table S4.** List of *Dnmt3* sequences used for computing dN/dS ratios. Sequences were downloaded from the NCBI Nucleotide database.

## Data Availability

The RNA-seq data sets supporting the conclusion of this article are available in the Gene Expression Omnibus (GEO) repository with accession number GSE236457. Custom scripts are available upon request.
