# Supplementary material for: Expression analysis suggests that DNMT3L is required for oocyte de novo DNA methylation only in Muridae and Cricetidae rodents
Source: Epigenetics Chromatin. 2023 Nov 4;16:43. doi: 10.1186/s13072-023-00518-2 (PMC10625200; doi:10.1186/s13072-023-00518-2)
Supplement: Supplementary file 5 — Additional file 5. Multiple sequence alignment of rodent Aire intron 3 sequences. [file 13072_2023_518_MOESM5_ESM.docx]

CLUSTAL multiple sequence alignment by MUSCLE (3.8)

ENSTBET00000016839.1 CTGCAGG----------GGATAGCTGGAGACAGCCTGGGGA------------CCAGTAG

ENSNGAT00000007851.1 CTGCAAA----------GGGGTGGCAGAGGCAGCCT----------GGCTCTCCTCTCTG

ENSMUGT00000023874.1 CTGGGAA----------AGGGAGGTGGAGGCAGGCT----------AGGCCTCCTTTCTG

ENSRNOT00000001611.5 CTGTGAA----------GGGGAGGTACAGG---------------------------TTG

MGP_CAROLIEiJ_T0023625.1 CTGCGAA----------GGGGAGGTAGAGGCAGCCTG---------GGCCCACTTTTCTG

ENSMUST00000128241.8 CTGCGAA----------GGGGAGGTAGAGGCAGCCTG---------GGCCCACTTTTCTG

ENSMSIT00000039370.1 CTGCGAA----------GGGGAGGTAGAGGCAGCCTG---------GGCCCACTTTTCTG

MGP_SPRETEiJ_T0024771.1 CTGCGAA----------GGGGAGGTAGAGGCAGCCTG---------GGCCCACTTTTCTG

ENSMAUT00000029410.1 CTGCCAA----------GGAGAGGTGGAGGCAGCCTGAAGGT----AAAGCC-CTTTCTG

ENSCGRT00001027434.1 CTGCTGA----------GGGGAGTTGGAAGCAGCATGAAGGCAATGAATGCC-CTTTCTG

ENSPEMT00000030353.2 CTGTGAAGTGGGGTGGGGGGGAAGTAGAGGCAGCCTGGAGGT----ATCCTC-CTTTCTG

ENSMOCT00000010482.1 CTGTGAA----------GGGGAGGTGGAGACAGCCTGAAGGT----ATCCTCTTTTTCTG

ENSCCNT00000026806.1 CTGCAAG----------GGGGAGCTAGAGGCAGC----------TGGGGCCTCCTCTCCT

ENSJJAT00000021205.1 CTGTAA-----------GGGGAGCTGGTGGCAGCCTG---------GTCCAGCTTCTGAG

ENSHGLT00000023656.1 CTGCAAA----------GGGGTGCTGGGGTAATC-------------------------T

ENSCLAT00000006778.1 CTGCACA----------GGGGTGCTGGGGGCAGT-------------------------G

ENSFDAT00000003362.1 CTGCAAA----------GGGGTACTGGGGTCAGC-------------------------T

ENSSVLT00005034202.1 CTGGAAG----------GGAGAGCTGGAGGCAGCTGGG-------------CCTCTGTGG

ENSSTOT00000021109.2 CTG-----------------GAGCCAGAAA------------------------------

ENSMMMT00000015955.1 CTGGAAG----------GGAGAGCTGGGAGCAG-------------GGGGCTTCGGCAGG

ENSUPAT00010004184.1 CTGGAAG----------GGAGAGCTGGGGGCAG-------------GGGGCTTCCACAGG

***

ENSTBET00000016839.1 GGCCAGGGTGGCCCAGGTGGAAT-------------------------------GGGGGC

ENSNGAT00000007851.1 G----AGGGGGTACTAGTGGGCC--AGGAAGTGGAGTGGGGTGGGG--------GACAGC

ENSMUGT00000023874.1 GGCTGGGGTGGTGCTGGTGGGCCC-AGGGTATAAAGTGAGGTGGGGGGGTGGGAGAAAGC

ENSRNOT00000001611.5 GGTTAGGG-GGTGTTGACAGGTCC-AGGATGTCAAGTAAGGTTGGG-GA-----GAAAGC

MGP_CAROLIEiJ_T0023625.1 GGCTGGGGTGGTGTTGATGGATCC-AGAGTAGAAAGTGAGGTTGGGAGA-----GAGAGC

ENSMUST00000128241.8 GGCTGGGGTCATGTTGACGGATCC-AGGGTAGAAAGTGAGGATGGGAGA-----GAGAGC

ENSMSIT00000039370.1 GGCTGGGGTCATGTTGACGGATCC-AGGGTAGAAAGTGAGGATGGGAGA-----GAGAGC

MGP_SPRETEiJ_T0024771.1 GGCTGGGGTCATGTTGGCGGATCC-AGGGTAGAAAGTGAGGATGGGAGA-----GAGAGC

ENSMAUT00000029410.1 GGCCGGGGCGGTCCTG-----------------------GGT-GG--GG-----GAGAGC

ENSCGRT00001027434.1 GGCTGGGGTGGTGTTGGTGGGTCC-AGGGTATAAAGTGAGGC-GG--GG-----GAGAGC

ENSPEMT00000030353.2 GGCTGGGGTGGTGCTGGCGGGTCCCAGGGTATAAAGTGAGGTGGG--GG-----GAGAGC

ENSMOCT00000010482.1 GGCTGGGGTGGTGCTGGTGGGTCC--AGGTCTCAAGTGAGATGGGG--------GAGAGC

ENSCCNT00000026806.1 GGCTAGGG-----CTGGTGGGCCT-GGTGTGTGTGGGGGAGGAGGATGG-----GGGAGA

ENSJJAT00000021205.1 GGCTGGTGCCAGGCCCAGGGGA--------------------------------TGGGGC

ENSHGLT00000023656.1 GGCTG-------------------------------------------------GAGAGC

ENSCLAT00000006778.1 GGCTG-------------------------------------------------GAGAGC

ENSFDAT00000003362.1 GGCTG-------------------------------------------------GGGAGC

ENSSVLT00005034202.1 GGCGGGGACAATGCTGGTGGCC---------------------GGGTGG-----GAAGGC

ENSSTOT00000021109.2 ------------------------------------------------------GGGGG-

ENSMMMT00000015955.1 GGCGGGGGCAATGCTGGTGGCC---------------------AGGTGG-----GAGGGC

ENSUPAT00010004184.1 GGCGGGGCAGCAGGTGG-------------------------------------GAGGAC

ENSTBET00000016839.1 ACAGGTTTAGT------------------CTTCCTCACTCTCTGTGGGTTCCTTTGCTTT

ENSNGAT00000007851.1 ATCTGGT-GGGG----TCAGATGCTGGGGTTCCCTGACCCCCTGTGAGTTTCTCCACTGG

ENSMUGT00000023874.1 ACCTGGC-AGGG----TCAGATCATGGG-TTCCCTGGCTCCCTGAGAGTTCTTAAGCTGG

ENSRNOT00000001611.5 ACCTGGTAAGGG----TCATATCAAGGG-TTCCCTGGATCCCTGAGGGTTCTTAAGCTGG

MGP_CAROLIEiJ_T0023625.1 AGCTGGTAAGGG----TCAGATCAAGGG-TTCCCTGACTCCCTGATGGTTCTTAAGCTGT

ENSMUST00000128241.8 AGCTGGTAAGGG----TCAGATC--------------------GACGGTTCTTAAGCTGT

ENSMSIT00000039370.1 AGCTGGTAAGGG----TCAGGTCAAGGG-TTCCCTGACTCCCTGAGGGTTCTTAAGCTGT

MGP_SPRETEiJ_T0024771.1 AGCTGGTAAGGG----TCAGGTCAAGGG-TTCCCTGACTCCCTGAGGGTTCTTAAGCTGT

ENSMAUT00000029410.1 ACCTGGT-AGGG----GCAGATCAAGGG-TTTCCTGACCCCCTGAGTGTTCTTAAGCCGT

ENSCGRT00001027434.1 ACCTGGT-AGGG----CCAGACCAAGGGATTTCCTGACCCCCTGAGTGTTCTTAAGCCAG

ENSPEMT00000030353.2 ACCTGGT-AGGG----TCAGATCAAGGG-TTCCCTGACTCCCTGAGGGTTCTTAAGCTGT

ENSMOCT00000010482.1 ACCTGGG-AGGG----TCAGATCAAGGG-TTCCCTAACTCCCT---------TCAGCCGT

ENSCCNT00000026806.1 GTTGGGG-GGTGTGGATTAGGTGCTGGG--------------------------------

ENSJJAT00000021205.1 ATTGGTG-TGAGAGGATCACCTGCAGA----TCCCAGCTCCCAGAGAGTTCCTTCAGCAT

ENSHGLT00000023656.1 ACTGGTGCGGTG-------GGTGCTGGG-GCTCTGGGCTCCCTGGGGGTCCCAGTG----

ENSCLAT00000006778.1 ACTGGGT-GGGG-----TGGGTGCTGGGGCTCCTGGGCTCTATGCAGACCCCTTTGCTGT

ENSFDAT00000003362.1 ACTGGGT-AGGG-----CAGGTGCTGGGTCTCCCGGCATCCCTGGGGG------------

ENSSVLT00005034202.1 ACCATGT-GGGG----TCAGGTGCTGG----CCCTGACTCCCTGTGGGCTCCTTCATGGT

ENSSTOT00000021109.2 --------GGGG------------------------------------------------

ENSMMMT00000015955.1 AGCAGGT-GGAG----TCAGGTGCTGG----CCCTGACTCCCTGTGGGCTCCTTCATGGT

ENSUPAT00010004184.1 AGCAGGT-GGAG----TCAGGTGCTGG----CCCTGACTCCCTGTTGGCTCCTTCGTGGT

*

ENSTBET00000016839.1 AGTGG---GTTCTGGTTTACTCACC-----------------------------------

ENSNGAT00000007851.1 TGGGA---GC--TGTTTCTTGCTTCAGTAAAAAGTCATTTACAGTAGACTGATTTTTTCA

ENSMUGT00000023874.1 CACAG---GC--TGTTTCCTTCTTC-----------------------------------

ENSRNOT00000001611.5 TCTGG---GCTGTGTTTCCTTCTTC-----------------------------------

MGP_CAROLIEiJ_T0023625.1 GATAG---GC--TGTTTCCTTCTTC-----------------------------------

ENSMUST00000128241.8 GATGG---GC--TGTTTCCTTCTTC-----------------------------------

ENSMSIT00000039370.1 GATGG---GC--TGTTTCCTTCTTC-----------------------------------

MGP_SPRETEiJ_T0024771.1 GATGG---GC--TGTTTCCTTCTTC-----------------------------------

ENSMAUT00000029410.1 GTTAG---GC--CATTTCCTTCTTC-----------------------------------

ENSCGRT00001027434.1 GCTAG---GC--CGTTTCCTTCTTC-----------------------------------

ENSPEMT00000030353.2 GCTAG---GC--TGCTTCCTTTTCA-----------------------------------

ENSMOCT00000010482.1 AGAGG------CTGTTTCCTTTTTC-----------------------------------

ENSCCNT00000026806.1 -----------CTATTTCCCTCAGT-----------------------------------

ENSJJAT00000021205.1 GCTAGGTTGTTCTGCTTCCTTCCTTTGTAAGTATTTTTATTTATTTATTTT---------

ENSHGLT00000023656.1 -------------GATCCCTTTGCC-----------------------------------

ENSCLAT00000006778.1 CAGGA-------------------------------------------------------

ENSFDAT00000003362.1 ------------------------------------------------------------

ENSSVLT00005034202.1 CCTGG---GCTCTGGTTTCCTCACC-----------------------------------

ENSSTOT00000021109.2 ------------------------------------------------------------

ENSMMMT00000015955.1 ACTGG---GCTCTGGTTTCCTCACC-----------------------------------

ENSUPAT00010004184.1 ACTGG---GCTCTGGTTTCCTCAGC-----------------------------------

ENSTBET00000016839.1 ------------------------------------------------------------

ENSNGAT00000007851.1 AGGTAATTTTAGCAATTTAAGAAGTATCTTGGATGGAAAGCAGGAAAAAAAAAAAAAAAA

ENSMUGT00000023874.1 ------------------------------------------------------------

ENSRNOT00000001611.5 ------------------------------------------------------------

MGP_CAROLIEiJ_T0023625.1 ------------------------------------------------------------

ENSMUST00000128241.8 ------------------------------------------------------------

ENSMSIT00000039370.1 ------------------------------------------------------------

MGP_SPRETEiJ_T0024771.1 ------------------------------------------------------------

ENSMAUT00000029410.1 ------------------------------------------------------------

ENSCGRT00001027434.1 ------------------------------------------------------------

ENSPEMT00000030353.2 ------------------------------------------------------------

ENSMOCT00000010482.1 ------------------------------------------------------------

ENSCCNT00000026806.1 ------------------------------------------------------------

ENSJJAT00000021205.1 ------------------------------------------------------------

ENSHGLT00000023656.1 ------------------------------------------------------------

ENSCLAT00000006778.1 ------------------------------------------------------------

ENSFDAT00000003362.1 ------------------------------------------------------------

ENSSVLT00005034202.1 ------------------------------------------------------------

ENSSTOT00000021109.2 ------------------------------------------------------------

ENSMMMT00000015955.1 ------------------------------------------------------------

ENSUPAT00010004184.1 ------------------------------------------------------------

ENSTBET00000016839.1 ----------AGTAC-AAAGTTAACAAAAG------------------------------

ENSNGAT00000007851.1 AAAAAAAAAAAAAAAAAAAGGCAGTTAAAA------------------------------

ENSMUGT00000023874.1 ----------AACAG-AAAGGAAATCAAAA------------------------------

ENSRNOT00000001611.5 ----------AACAGAAAAGGAAATCAAAA------------------------------

MGP_CAROLIEiJ_T0023625.1 ----------AACGG-AAAGGAAATCAAAA------------------------------

ENSMUST00000128241.8 ----------AACAG-AAAGGAAATCAAAA------------------------------

ENSMSIT00000039370.1 ----------AACAG-AAAGGAAATCAAAA------------------------------

MGP_SPRETEiJ_T0024771.1 ----------AACAG-AAAGGAAATCAAAA------------------------------

ENSMAUT00000029410.1 ----------AACAG-AAACGAAATCAAAA------------------------------

ENSCGRT00001027434.1 ----------AACGG-AAATGAAATCAAAA------------------------------

ENSPEMT00000030353.2 ----------ATGGG-AAACGAAATCAAAA------------------------------

ENSMOCT00000010482.1 ----------AGCGG-AAACGAAATCAAAA------------------------------

ENSCCNT00000026806.1 ----------AGTAAAAAGGAAAGAAACCA------------------------------

ENSJJAT00000021205.1 ----------TACAGAGAGAGAAAGAAACAGATAAAGAGAGACAGAGAATGGGTGGGCCA

ENSHGLT00000023656.1 ----------GCTGGGATCTGGAACCCCGG------------------------------

ENSCLAT00000006778.1 ----------------ACAGGAAACCCCAC------------------------------

ENSFDAT00000003362.1 ------------------------------------------------------------

ENSSVLT00005034202.1 ----------AGTAAAAAGGAAAATGAGAA------------------------------

ENSSTOT00000021109.2 ------------------------------------------------------------

ENSMMMT00000015955.1 ----------AGTAGAAAGGAAAATGAGAC------------------------------

ENSUPAT00010004184.1 ----------AGTAGAAAGGAAAATGAGA-------------------------------

ENSTBET00000016839.1 -----------------------------------------CCACCTACCTACTT----C

ENSNGAT00000007851.1 -----------------------------------------CCACCTGCCTGCCC----C

ENSMUGT00000023874.1 -----------------------------------------GCACCTGCCTGCCT----C

ENSRNOT00000001611.5 -----------------------------------------CCACCTGCCTGCCTCACCC

MGP_CAROLIEiJ_T0023625.1 -----------------------------------------CCACCTGCCTGCCT----C

ENSMUST00000128241.8 -----------------------------------------CCACCTGCCTGCCT----C

ENSMSIT00000039370.1 -----------------------------------------CCACCTGCCTGCCT----C

MGP_SPRETEiJ_T0024771.1 -----------------------------------------CCACCTGCCTGCCT----C

ENSMAUT00000029410.1 -----------------------------------------CCACCTGCCTGCCT----C

ENSCGRT00001027434.1 -----------------------------------------CCACCTGCCTGCCT----C

ENSPEMT00000030353.2 -----------------------------------------CCACCTGCCTGCCT----C

ENSMOCT00000010482.1 -----------------------------------------CCACCTGCCTGCCT----C

ENSCCNT00000026806.1 -----------------------------------------CTCCCTGGGTGGTG-----

ENSJJAT00000021205.1 GGGTCTCCAGCCACTGCAGATGAACTCCAGAAGTATGCCCCCCCCTTGTGCATCTGGCTA

ENSHGLT00000023656.1 -----------------------------------------CCACCTGGGCTCCTGGGCC

ENSCLAT00000006778.1 -----------------------------------------CCACCTGGGCACCTGGGCC

ENSFDAT00000003362.1 -----------------------------------------CCCCCTGTGGA-------A

ENSSVLT00005034202.1 -----------------------------------------CTGCCTGCCCCTTT-----

ENSSTOT00000021109.2 -----------------------------------------ACCCCT-------------

ENSMMMT00000015955.1 -----------------------------------------CCTCCTGCCCCTTT-----

ENSUPAT00010004184.1 -----------------------------------------CTCCCTGCCCCTTT-----

* *

ENSTBET00000016839.1 ACA--GCCCCTGGGGAGCTCAGGGT-----------------------------------

ENSNGAT00000007851.1 ACC--GCCC---AGGAACTCAGCCT-----------------------------------

ENSMUGT00000023874.1 ACC--ACGA---AGGAACTCGGCCT-----------------------------------

ENSRNOT00000001611.5 ACC--ACCC---TGGAACCCAGCCT-----------------------------------

MGP_CAROLIEiJ_T0023625.1 ACC--ACCC---AGGAACTCAGCCT-----------------------------------

ENSMUST00000128241.8 ACC--GCCC---AGAAACTCAGCCT-----------------------------------

ENSMSIT00000039370.1 ACC--GCCC---AGGAACTCAGCCT-----------------------------------

MGP_SPRETEiJ_T0024771.1 ACC--GCCC---AGGAACTCAGCCT-----------------------------------

ENSMAUT00000029410.1 ACC--AGCCAGGAGGAACTCAGCCT-----------------------------------

ENSCGRT00001027434.1 ACC--AGCC---AGGAACTCAGCCT-----------------------------------

ENSPEMT00000030353.2 ACC--ACCC---AGGAACTCAGCCT-----------------------------------

ENSMOCT00000010482.1 ACT--ACCC---AGGAACTCAGTCT-----------------------------------

ENSCCNT00000026806.1 -------ACCCAGGGAATTCTGCCT-----------------------------------

ENSJJAT00000021205.1 ACGTGGGTCCTGGGGAATTGAGCCTCAAACTGGGGTCTTTAGCTTCACAGGCAAGTGCTT

ENSHGLT00000023656.1 GCCTGGGCGCTGGGCAGCTGGGC-------------------------------------

ENSCLAT00000006778.1 ACC-----------TCACCCAGCCT-----------------------------------

ENSFDAT00000003362.1 ACCCCAAGCCCTGGGATCCTGGCCT-----------------------------------

ENSSVLT00005034202.1 -----AGCGCTTAAGGACTCAGTGTTGG--------------------------------

ENSSTOT00000021109.2 ------------------------------------------------------------

ENSMMMT00000015955.1 -----ACCACTTAAAAACTCAGAC------------------------------------

ENSUPAT00010004184.1 -----ACCACTTAAAAACTCAGACC-----------------------------------

ENSTBET00000016839.1 ------------------------------------------------------------

ENSNGAT00000007851.1 ------------------------------------------------------------

ENSMUGT00000023874.1 ------------------------------------------------------------

ENSRNOT00000001611.5 ------------------------------------------------------------

MGP_CAROLIEiJ_T0023625.1 ------------------------------------------------------------

ENSMUST00000128241.8 ------------------------------------------------------------

ENSMSIT00000039370.1 ------------------------------------------------------------

MGP_SPRETEiJ_T0024771.1 ------------------------------------------------------------

ENSMAUT00000029410.1 ------------------------------------------------------------

ENSCGRT00001027434.1 ------------------------------------------------------------

ENSPEMT00000030353.2 ------------------------------------------------------------

ENSMOCT00000010482.1 ------------------------------------------------------------

ENSCCNT00000026806.1 ------------------------------------------------------------

ENSJJAT00000021205.1 AATCCTAAGCCATATCTCCAGCACAATATTTTTTATTTATTTATTTATTTATTTATTTGA

ENSHGLT00000023656.1 ------------------------------------------------------------

ENSCLAT00000006778.1 ------------------------------------------------------------

ENSFDAT00000003362.1 ------------------------------------------------------------

ENSSVLT00005034202.1 ------------------------------------------------------------

ENSSTOT00000021109.2 ------------------------------------------------------------

ENSMMMT00000015955.1 ------------------------------------------------------------

ENSUPAT00010004184.1 ------------------------------------------------------------

ENSTBET00000016839.1 ------------------------------------------------------------

ENSNGAT00000007851.1 ------------------------------------------------------------

ENSMUGT00000023874.1 ------------------------------------------------------------

ENSRNOT00000001611.5 ------------------------------------------------------------

MGP_CAROLIEiJ_T0023625.1 ------------------------------------------------------------

ENSMUST00000128241.8 ------------------------------------------------------------

ENSMSIT00000039370.1 ------------------------------------------------------------

MGP_SPRETEiJ_T0024771.1 ------------------------------------------------------------

ENSMAUT00000029410.1 ------------------------------------------------------------

ENSCGRT00001027434.1 ------------------------------------------------------------

ENSPEMT00000030353.2 ------------------------------------------------------------

ENSMOCT00000010482.1 ------------------------------------------------------------

ENSCCNT00000026806.1 ------------------------------------------------------------

ENSJJAT00000021205.1 AAGAGAGAAAAAGGGGTAGAGTGTATGTGTGTGCCAGGGCCTCCAGCCACTGCAAACAAA

ENSHGLT00000023656.1 ------------------------------------------------------------

ENSCLAT00000006778.1 ------------------------------------------------------------

ENSFDAT00000003362.1 ------------------------------------------------------------

ENSSVLT00005034202.1 ------------------------------------------------------------

ENSSTOT00000021109.2 ------------------------------------------------------------

ENSMMMT00000015955.1 ------------------------------------------------------------

ENSUPAT00010004184.1 ------------------------------------------------------------

ENSTBET00000016839.1 ------------------------------------------------------------

ENSNGAT00000007851.1 ------------------------------------------------------------

ENSMUGT00000023874.1 ------------------------------------------------------------

ENSRNOT00000001611.5 ------------------------------------------------------------

MGP_CAROLIEiJ_T0023625.1 ------------------------------------------------------------

ENSMUST00000128241.8 ------------------------------------------------------------

ENSMSIT00000039370.1 ------------------------------------------------------------

MGP_SPRETEiJ_T0024771.1 ------------------------------------------------------------

ENSMAUT00000029410.1 ------------------------------------------------------------

ENSCGRT00001027434.1 ------------------------------------------------------------

ENSPEMT00000030353.2 ------------------------------------------------------------

ENSMOCT00000010482.1 ------------------------------------------------------------

ENSCCNT00000026806.1 ------------------------------------------------------------

ENSJJAT00000021205.1 CTCCAGATGCATGCATCCCCTTCTGCATCTGGCTTACATCGGTCCTGGAGAATCAAACTA

ENSHGLT00000023656.1 ------------------------------------------------------------

ENSCLAT00000006778.1 ------------------------------------------------------------

ENSFDAT00000003362.1 ------------------------------------------------------------

ENSSVLT00005034202.1 ------------------------------------------------------------

ENSSTOT00000021109.2 ------------------------------------------------------------

ENSMMMT00000015955.1 ------------------------------------------------------------

ENSUPAT00010004184.1 ------------------------------------------------------------

ENSTBET00000016839.1 ------------------------------------------------------------

ENSNGAT00000007851.1 ------------------------------------------------------------

ENSMUGT00000023874.1 ------------------------------------------------------------

ENSRNOT00000001611.5 ------------------------------------------------------------

MGP_CAROLIEiJ_T0023625.1 ------------------------------------------------------------

ENSMUST00000128241.8 ------------------------------------------------------------

ENSMSIT00000039370.1 ------------------------------------------------------------

MGP_SPRETEiJ_T0024771.1 ------------------------------------------------------------

ENSMAUT00000029410.1 ------------------------------------------------------------

ENSCGRT00001027434.1 ------------------------------------------------------------

ENSPEMT00000030353.2 ------------------------------------------------------------

ENSMOCT00000010482.1 ------------------------------------------------------------

ENSCCNT00000026806.1 ------------------------------------------------------------

ENSJJAT00000021205.1 GCATCCTTTGGCTTTGAAGGCAAAGGCCTTAACCACCAAGCCATCGCTCCAGCCCCTAAA

ENSHGLT00000023656.1 ------------------------------------------------------------

ENSCLAT00000006778.1 ------------------------------------------------------------

ENSFDAT00000003362.1 ------------------------------------------------------------

ENSSVLT00005034202.1 ------------------------------------------------------------

ENSSTOT00000021109.2 ------------------------------------------------------------

ENSMMMT00000015955.1 ------------------------------------------------------------

ENSUPAT00010004184.1 ------------------------------------------------------------

ENSTBET00000016839.1 ------------------------------------------------------------

ENSNGAT00000007851.1 ------------------------------------------------------------

ENSMUGT00000023874.1 ------------------------------------------------------------

ENSRNOT00000001611.5 ------------------------------------------------------------

MGP_CAROLIEiJ_T0023625.1 ------------------------------------------------------------

ENSMUST00000128241.8 ------------------------------------------------------------

ENSMSIT00000039370.1 ------------------------------------------------------------

MGP_SPRETEiJ_T0024771.1 ------------------------------------------------------------

ENSMAUT00000029410.1 ------------------------------------------------------------

ENSCGRT00001027434.1 ------------------------------------------------------------

ENSPEMT00000030353.2 ------------------------------------------------------------

ENSMOCT00000010482.1 ------------------------------------------------------------

ENSCCNT00000026806.1 ------------------------------------------------------------

ENSJJAT00000021205.1 TATTTTTATTTATTTGCAAGGAGTTGGGGGAGGGGAGAATATGAGCATGTGTGGGCCTCT

ENSHGLT00000023656.1 ------------------------------------------------------------

ENSCLAT00000006778.1 ------------------------------------------------------------

ENSFDAT00000003362.1 ------------------------------------------------------------

ENSSVLT00005034202.1 ------------------------------------------------------------

ENSSTOT00000021109.2 ------------------------------------------------------------

ENSMMMT00000015955.1 ------------------------------------------------------------

ENSUPAT00010004184.1 ------------------------------------------------------------

ENSTBET00000016839.1 ------------------------------------------------------------

ENSNGAT00000007851.1 ------------------------------------------------------------

ENSMUGT00000023874.1 ------------------------------------------------------------

ENSRNOT00000001611.5 ------------------------------------------------------------

MGP_CAROLIEiJ_T0023625.1 ------------------------------------------------------------

ENSMUST00000128241.8 ------------------------------------------------------------

ENSMSIT00000039370.1 ------------------------------------------------------------

MGP_SPRETEiJ_T0024771.1 ------------------------------------------------------------

ENSMAUT00000029410.1 ------------------------------------------------------------

ENSCGRT00001027434.1 ------------------------------------------------------------

ENSPEMT00000030353.2 ------------------------------------------------------------

ENSMOCT00000010482.1 ------------------------------------------------------------

ENSCCNT00000026806.1 ------------------------------------------------------------

ENSJJAT00000021205.1 TGCCACTGCAAATGAACTCCAGAGGCATGTGCCACTTTGGGCATCTTGCTTTCGTGGGTC

ENSHGLT00000023656.1 ------------------------------------------------------------

ENSCLAT00000006778.1 ------------------------------------------------------------

ENSFDAT00000003362.1 ------------------------------------------------------------

ENSSVLT00005034202.1 ------------------------------------------------------------

ENSSTOT00000021109.2 ------------------------------------------------------------

ENSMMMT00000015955.1 ------------------------------------------------------------

ENSUPAT00010004184.1 ------------------------------------------------------------

ENSTBET00000016839.1 ------------------------------------------------------------

ENSNGAT00000007851.1 ------------------------------------------------------------

ENSMUGT00000023874.1 ------------------------------------------------------------

ENSRNOT00000001611.5 ------------------------------------------------------------

MGP_CAROLIEiJ_T0023625.1 ------------------------------------------------------------

ENSMUST00000128241.8 ------------------------------------------------------------

ENSMSIT00000039370.1 ------------------------------------------------------------

MGP_SPRETEiJ_T0024771.1 ------------------------------------------------------------

ENSMAUT00000029410.1 ------------------------------------------------------------

ENSCGRT00001027434.1 ------------------------------------------------------------

ENSPEMT00000030353.2 ------------------------------------------------------------

ENSMOCT00000010482.1 ------------------------------------------------------------

ENSCCNT00000026806.1 ------------------------------------------------------------

ENSJJAT00000021205.1 CTGAGGAATCGAACCCAAGCTGTCAGGCTTTGCAAGTAAGTGCCTTTAACCGCTGAGCCA

ENSHGLT00000023656.1 ------------------------------------------------------------

ENSCLAT00000006778.1 ------------------------------------------------------------

ENSFDAT00000003362.1 ------------------------------------------------------------

ENSSVLT00005034202.1 ------------------------------------------------------------

ENSSTOT00000021109.2 ------------------------------------------------------------

ENSMMMT00000015955.1 ------------------------------------------------------------

ENSUPAT00010004184.1 ------------------------------------------------------------

ENSTBET00000016839.1 ------------------------------------------------------------

ENSNGAT00000007851.1 ------------------------------------------------------------

ENSMUGT00000023874.1 ------------------------------------------------------------

ENSRNOT00000001611.5 ------------------------------------------------------------

MGP_CAROLIEiJ_T0023625.1 ------------------------------------------------------------

ENSMUST00000128241.8 ------------------------------------------------------------

ENSMSIT00000039370.1 ------------------------------------------------------------

MGP_SPRETEiJ_T0024771.1 ------------------------------------------------------------

ENSMAUT00000029410.1 ------------------------------------------------------------

ENSCGRT00001027434.1 ------------------------------------------------------------

ENSPEMT00000030353.2 ------------------------------------------------------------

ENSMOCT00000010482.1 ------------------------------------------------------------

ENSCCNT00000026806.1 ------------------------------------------------------------

ENSJJAT00000021205.1 TGTCTCCAGCACCTTTCACTTTATATAGATGATGGAGAGATGGCTCAGCGGTTAGGCACT

ENSHGLT00000023656.1 ------------------------------------------------------------

ENSCLAT00000006778.1 ------------------------------------------------------------

ENSFDAT00000003362.1 ------------------------------------------------------------

ENSSVLT00005034202.1 ------------------------------------------------------------

ENSSTOT00000021109.2 ------------------------------------------------------------

ENSMMMT00000015955.1 ------------------------------------------------------------

ENSUPAT00010004184.1 ------------------------------------------------------------

ENSTBET00000016839.1 ------------------------------------------------------------

ENSNGAT00000007851.1 ------------------------------------------------------------

ENSMUGT00000023874.1 ------------------------------------------------------------

ENSRNOT00000001611.5 ------------------------------------------------------------

MGP_CAROLIEiJ_T0023625.1 ------------------------------------------------------------

ENSMUST00000128241.8 ------------------------------------------------------------

ENSMSIT00000039370.1 ------------------------------------------------------------

MGP_SPRETEiJ_T0024771.1 ------------------------------------------------------------

ENSMAUT00000029410.1 ------------------------------------------------------------

ENSCGRT00001027434.1 ------------------------------------------------------------

ENSPEMT00000030353.2 ------------------------------------------------------------

ENSMOCT00000010482.1 ------------------------------------------------------------

ENSCCNT00000026806.1 ------------------------------------------------------------

ENSJJAT00000021205.1 TACCTGCAAAGCTTAAGGACCCAAGTTCAATTTCCCAGGATCCATGTAAGCCAGATGCAC

ENSHGLT00000023656.1 ------------------------------------------------------------

ENSCLAT00000006778.1 ------------------------------------------------------------

ENSFDAT00000003362.1 ------------------------------------------------------------

ENSSVLT00005034202.1 ------------------------------------------------------------

ENSSTOT00000021109.2 ------------------------------------------------------------

ENSMMMT00000015955.1 ------------------------------------------------------------

ENSUPAT00010004184.1 ------------------------------------------------------------

ENSTBET00000016839.1 ------------------------------------------------------------

ENSNGAT00000007851.1 ------------------------------------------------------------

ENSMUGT00000023874.1 ------------------------------------------------------------

ENSRNOT00000001611.5 ------------------------------------------------------------

MGP_CAROLIEiJ_T0023625.1 ------------------------------------------------------------

ENSMUST00000128241.8 ------------------------------------------------------------

ENSMSIT00000039370.1 ------------------------------------------------------------

MGP_SPRETEiJ_T0024771.1 ------------------------------------------------------------

ENSMAUT00000029410.1 ------------------------------------------------------------

ENSCGRT00001027434.1 ------------------------------------------------------------

ENSPEMT00000030353.2 ------------------------------------------------------------

ENSMOCT00000010482.1 ------------------------------------------------------------

ENSCCNT00000026806.1 ------------------------------------------------------------

ENSJJAT00000021205.1 AAGGTGGCCCATGCAGCTGAAGTTACTTTGTTGTGACTGGAGGACTTGGCATGCCCATCC

ENSHGLT00000023656.1 ------------------------------------------------------------

ENSCLAT00000006778.1 ------------------------------------------------------------

ENSFDAT00000003362.1 ------------------------------------------------------------

ENSSVLT00005034202.1 ------------------------------------------------------------

ENSSTOT00000021109.2 ------------------------------------------------------------

ENSMMMT00000015955.1 ------------------------------------------------------------

ENSUPAT00010004184.1 ------------------------------------------------------------

ENSTBET00000016839.1 ------------------------------------------------------------

ENSNGAT00000007851.1 ------------------------------------------------------------

ENSMUGT00000023874.1 ------------------------------------------------------------

ENSRNOT00000001611.5 ------------------------------------------------------------

MGP_CAROLIEiJ_T0023625.1 ------------------------------------------------------------

ENSMUST00000128241.8 ------------------------------------------------------------

ENSMSIT00000039370.1 ------------------------------------------------------------

MGP_SPRETEiJ_T0024771.1 ------------------------------------------------------------

ENSMAUT00000029410.1 ------------------------------------------------------------

ENSCGRT00001027434.1 ------------------------------------------------------------

ENSPEMT00000030353.2 ------------------------------------------------------------

ENSMOCT00000010482.1 ------------------------------------------------------------

ENSCCNT00000026806.1 ------------------------------------------------------------

ENSJJAT00000021205.1 AATCTCTCTCTCTCTCTCTGTCTGCCTCTTCTTCTCTTTTTCCCTTGTCCCTCTCATATA

ENSHGLT00000023656.1 ------------------------------------------------------------

ENSCLAT00000006778.1 ------------------------------------------------------------

ENSFDAT00000003362.1 ------------------------------------------------------------

ENSSVLT00005034202.1 ------------------------------------------------------------

ENSSTOT00000021109.2 ------------------------------------------------------------

ENSMMMT00000015955.1 ------------------------------------------------------------

ENSUPAT00010004184.1 ------------------------------------------------------------

ENSTBET00000016839.1 ------------------------------------------------------------

ENSNGAT00000007851.1 ------------------------------------------------------------

ENSMUGT00000023874.1 ------------------------------------------------------------

ENSRNOT00000001611.5 ------------------------------------------------------------

MGP_CAROLIEiJ_T0023625.1 ------------------------------------------------------------

ENSMUST00000128241.8 ------------------------------------------------------------

ENSMSIT00000039370.1 ------------------------------------------------------------

MGP_SPRETEiJ_T0024771.1 ------------------------------------------------------------

ENSMAUT00000029410.1 ------------------------------------------------------------

ENSCGRT00001027434.1 ------------------------------------------------------------

ENSPEMT00000030353.2 ------------------------------------------------------------

ENSMOCT00000010482.1 ------------------------------------------------------------

ENSCCNT00000026806.1 ------------------------------------------------------------

ENSJJAT00000021205.1 TATATATATGGTTTTTTGAGGTAGGGTCTCGCTCTAGCCCAGGCTGACCTGGAGTTCACT

ENSHGLT00000023656.1 ------------------------------------------------------------

ENSCLAT00000006778.1 ------------------------------------------------------------

ENSFDAT00000003362.1 ------------------------------------------------------------

ENSSVLT00005034202.1 ------------------------------------------------------------

ENSSTOT00000021109.2 ------------------------------------------------------------

ENSMMMT00000015955.1 ------------------------------------------------------------

ENSUPAT00010004184.1 ------------------------------------------------------------

ENSTBET00000016839.1 ------------------------------------------------------------

ENSNGAT00000007851.1 ------------------------------------------------------------

ENSMUGT00000023874.1 ------------------------------------------------------------

ENSRNOT00000001611.5 ------------------------------------------------------------

MGP_CAROLIEiJ_T0023625.1 ------------------------------------------------------------

ENSMUST00000128241.8 ------------------------------------------------------------

ENSMSIT00000039370.1 ------------------------------------------------------------

MGP_SPRETEiJ_T0024771.1 ------------------------------------------------------------

ENSMAUT00000029410.1 ------------------------------------------------------------

ENSCGRT00001027434.1 ------------------------------------------------------------

ENSPEMT00000030353.2 ------------------------------------------------------------

ENSMOCT00000010482.1 ------------------------------------------------------------

ENSCCNT00000026806.1 ------------------------------------------------------------

ENSJJAT00000021205.1 ATGTAGTCTCAGGGTGACCTCAAACTCTTGGCAATCCTCCTATCTCTGCCTCTTGAGTGC

ENSHGLT00000023656.1 ------------------------------------------------------------

ENSCLAT00000006778.1 ------------------------------------------------------------

ENSFDAT00000003362.1 ------------------------------------------------------------

ENSSVLT00005034202.1 ------------------------------------------------------------

ENSSTOT00000021109.2 ------------------------------------------------------------

ENSMMMT00000015955.1 ------------------------------------------------------------

ENSUPAT00010004184.1 ------------------------------------------------------------

ENSTBET00000016839.1 -----------------------------------------------------------T

ENSNGAT00000007851.1 -----------------------------------------------------------T

ENSMUGT00000023874.1 -----------------------------------------------------------T

ENSRNOT00000001611.5 -----------------------------------------------------------T

MGP_CAROLIEiJ_T0023625.1 -----------------------------------------------------------T

ENSMUST00000128241.8 -----------------------------------------------------------T

ENSMSIT00000039370.1 -----------------------------------------------------------T

MGP_SPRETEiJ_T0024771.1 -----------------------------------------------------------T

ENSMAUT00000029410.1 -----------------------------------------------------------T

ENSCGRT00001027434.1 -----------------------------------------------------------T

ENSPEMT00000030353.2 -----------------------------------------------------------T

ENSMOCT00000010482.1 -----------------------------------------------------------G

ENSCCNT00000026806.1 ------------------------------------------------------------

ENSJJAT00000021205.1 TGGGATTATAGGTGTGCACCGCACCACCACGCCTGGCTTAAAAAAATGTCATTTATTTGC

ENSHGLT00000023656.1 ------------------------------------------------------------

ENSCLAT00000006778.1 -----------------------------------------------------------C

ENSFDAT00000003362.1 --------------------------------------------------------TGGG

ENSSVLT00005034202.1 ------------------------------------------------------GGCTGG

ENSSTOT00000021109.2 ------------------------------------------------------------

ENSMMMT00000015955.1 ------------------------------------------------------------

ENSUPAT00010004184.1 ------------------------------------------------------------

ENSTBET00000016839.1 TGGGACAGGACAGGGTTGGG---AGGATACG-----------------------------

ENSNGAT00000007851.1 CAGAGCAGGGAGGCGCTGAG---AGGCTGGG---A-------------------------

ENSMUGT00000023874.1 CAGACCAGTGAGAAGCTGAG---AGGCCGTG--AG-------------------------

ENSRNOT00000001611.5 CAGGACAGTAAGACGCTGAG---AGGCTGTGCCCT-------------------------

MGP_CAROLIEiJ_T0023625.1 CGGGACAGTAAGTCGCTGAG---AGGCTGTG--GG-------------------------

ENSMUST00000128241.8 TGGGACAGTAAGACGCTGAG---AGGCTGTG--GG-------------------------

ENSMSIT00000039370.1 TGGGACAGTAAGACGCTGAG---AGGCTGTG--GG-------------------------

MGP_SPRETEiJ_T0024771.1 TGGGACAGTAAGACGCTGAG---AGGCTGTG--GG-------------------------

ENSMAUT00000029410.1 CAGAACAGGAAGAAGCTGCG---AGGCGGTG--AG-------------------------

ENSCGRT00001027434.1 CAGAGCAGGAAGAAGCTGAG---AGGTACTG--AG-------------------------

ENSPEMT00000030353.2 CAGAACAGGAAGAAGCTGAG---AGGCGGTG--AG-------------------------

ENSMOCT00000010482.1 CAGAACAGGAAGAAGCTGCA---AGACTGTG--AG-------------------------

ENSCCNT00000026806.1 GAGGGTAGAAAGCAGCCAAG---GGCCGACA-----------------------------

ENSJJAT00000021205.1 AAGAAGAGAGAGAATTTGAA---TGGGTGTGTCAGGGCTTCTAGCCACTGCAAAGAGATT

ENSHGLT00000023656.1 -GGGAGAGGCAGGAGCCTGGCCCAGCCCGTG-----------------------------

ENSCLAT00000006778.1 CTGGACACCTGGGAGCCTGG---TGGCTGGG-----------------------------

ENSFDAT00000003362.1 CAGAAGAGGCAGGAGT--------------------------------------------

ENSSVLT00005034202.1 CACAGCTGACAGACCCGTCC---AGGCTGTC-----------------------------

ENSSTOT00000021109.2 ------------------------------------------------------------

ENSMMMT00000015955.1 ----------AGACCCTTCC---AGGGTATG-----------------------------

ENSUPAT00010004184.1 ---------------TGTCC---AGGGTATG-----------------------------

ENSTBET00000016839.1 ------------------------------------------------------------

ENSNGAT00000007851.1 ------------------------------------------------------------

ENSMUGT00000023874.1 ------------------------------------------------------------

ENSRNOT00000001611.5 ------------------------------------------------------------

MGP_CAROLIEiJ_T0023625.1 ------------------------------------------------------------

ENSMUST00000128241.8 ------------------------------------------------------------

ENSMSIT00000039370.1 ------------------------------------------------------------

MGP_SPRETEiJ_T0024771.1 ------------------------------------------------------------

ENSMAUT00000029410.1 ------------------------------------------------------------

ENSCGRT00001027434.1 ------------------------------------------------------------

ENSPEMT00000030353.2 ------------------------------------------------------------

ENSMOCT00000010482.1 ------------------------------------------------------------

ENSCCNT00000026806.1 ------------------------------------------------------------

ENSJJAT00000021205.1 TATGCACCACTTTGTGAATCTGGCTCTACAAGGGTACTGGGGAAAGGAACCCAGGTCATG

ENSHGLT00000023656.1 ------------------------------------------------------------

ENSCLAT00000006778.1 ------------------------------------------------------------

ENSFDAT00000003362.1 ------------------------------------------------------------

ENSSVLT00005034202.1 ------------------------------------------------------------

ENSSTOT00000021109.2 ------------------------------------------------------------

ENSMMMT00000015955.1 ------------------------------------------------------------

ENSUPAT00010004184.1 ------------------------------------------------------------

ENSTBET00000016839.1 ------------------------------------------------------------

ENSNGAT00000007851.1 ------------------------------------------------------------

ENSMUGT00000023874.1 ------------------------------------------------------------

ENSRNOT00000001611.5 ------------------------------------------------------------

MGP_CAROLIEiJ_T0023625.1 ------------------------------------------------------------

ENSMUST00000128241.8 ------------------------------------------------------------

ENSMSIT00000039370.1 ------------------------------------------------------------

MGP_SPRETEiJ_T0024771.1 ------------------------------------------------------------

ENSMAUT00000029410.1 ------------------------------------------------------------

ENSCGRT00001027434.1 ------------------------------------------------------------

ENSPEMT00000030353.2 ------------------------------------------------------------

ENSMOCT00000010482.1 ------------------------------------------------------------

ENSCCNT00000026806.1 ------------------------------------------------------------

ENSJJAT00000021205.1 AGGCTCTGCAGACAAGTGCCTCAACAGCTGAGCCATCTCTTCAGCTCCCCCACCCCTTCT

ENSHGLT00000023656.1 ------------------------------------------------------------

ENSCLAT00000006778.1 ------------------------------------------------------------

ENSFDAT00000003362.1 ------------------------------------------------------------

ENSSVLT00005034202.1 ------------------------------------------------------------

ENSSTOT00000021109.2 ------------------------------------------------------------

ENSMMMT00000015955.1 ------------------------------------------------------------

ENSUPAT00010004184.1 ------------------------------------------------------------

ENSTBET00000016839.1 ------------------------------------------------------------

ENSNGAT00000007851.1 ------------------------------------------------------------

ENSMUGT00000023874.1 ------------------------------------------------------------

ENSRNOT00000001611.5 ------------------------------------------------------------

MGP_CAROLIEiJ_T0023625.1 ------------------------------------------------------------

ENSMUST00000128241.8 ------------------------------------------------------------

ENSMSIT00000039370.1 ------------------------------------------------------------

MGP_SPRETEiJ_T0024771.1 ------------------------------------------------------------

ENSMAUT00000029410.1 ------------------------------------------------------------

ENSCGRT00001027434.1 ------------------------------------------------------------

ENSPEMT00000030353.2 ------------------------------------------------------------

ENSMOCT00000010482.1 ------------------------------------------------------------

ENSCCNT00000026806.1 ------------------------------------------------------------

ENSJJAT00000021205.1 TTTTTTAATGTGAGAAAGCATGAGTGAGAGAGAGAATTGGTGGCCAGGGTCTCCAGCCAC

ENSHGLT00000023656.1 ------------------------------------------------------------

ENSCLAT00000006778.1 ------------------------------------------------------------

ENSFDAT00000003362.1 ------------------------------------------------------------

ENSSVLT00005034202.1 ------------------------------------------------------------

ENSSTOT00000021109.2 ------------------------------------------------------------

ENSMMMT00000015955.1 ------------------------------------------------------------

ENSUPAT00010004184.1 ------------------------------------------------------------

ENSTBET00000016839.1 ------------------------------------------------------------

ENSNGAT00000007851.1 ------------------------------------------------------------

ENSMUGT00000023874.1 ------------------------------------------------------------

ENSRNOT00000001611.5 ------------------------------------------------------------

MGP_CAROLIEiJ_T0023625.1 ------------------------------------------------------------

ENSMUST00000128241.8 ------------------------------------------------------------

ENSMSIT00000039370.1 ------------------------------------------------------------

MGP_SPRETEiJ_T0024771.1 ------------------------------------------------------------

ENSMAUT00000029410.1 ------------------------------------------------------------

ENSCGRT00001027434.1 ------------------------------------------------------------

ENSPEMT00000030353.2 ------------------------------------------------------------

ENSMOCT00000010482.1 ------------------------------------------------------------

ENSCCNT00000026806.1 ------------------------------------------------------------

ENSJJAT00000021205.1 TGCAGTCGAACTCCCAACGCGTGCCCCACCTTGTGCGCATGTGCGACCGTGCGCACCTGC

ENSHGLT00000023656.1 ------------------------------------------------------------

ENSCLAT00000006778.1 ------------------------------------------------------------

ENSFDAT00000003362.1 ------------------------------------------------------------

ENSSVLT00005034202.1 ------------------------------------------------------------

ENSSTOT00000021109.2 ------------------------------------------------------------

ENSMMMT00000015955.1 ------------------------------------------------------------

ENSUPAT00010004184.1 ------------------------------------------------------------

ENSTBET00000016839.1 ------------------------------------------------------------

ENSNGAT00000007851.1 ------------------------------------------------------------

ENSMUGT00000023874.1 ------------------------------------------------------------

ENSRNOT00000001611.5 ------------------------------------------------------------

MGP_CAROLIEiJ_T0023625.1 ------------------------------------------------------------

ENSMUST00000128241.8 ------------------------------------------------------------

ENSMSIT00000039370.1 ------------------------------------------------------------

MGP_SPRETEiJ_T0024771.1 ------------------------------------------------------------

ENSMAUT00000029410.1 ------------------------------------------------------------

ENSCGRT00001027434.1 ------------------------------------------------------------

ENSPEMT00000030353.2 ------------------------------------------------------------

ENSMOCT00000010482.1 ------------------------------------------------------------

ENSCCNT00000026806.1 ------------------------------------------------------------

ENSJJAT00000021205.1 GTCACTTTGTGCTTGAGGCTTATGCGGGAACTGGCGCGTCAAACATGGGTCCTTAGGCTT

ENSHGLT00000023656.1 ------------------------------------------------------------

ENSCLAT00000006778.1 ------------------------------------------------------------

ENSFDAT00000003362.1 ------------------------------------------------------------

ENSSVLT00005034202.1 ------------------------------------------------------------

ENSSTOT00000021109.2 ------------------------------------------------------------

ENSMMMT00000015955.1 ------------------------------------------------------------

ENSUPAT00010004184.1 ------------------------------------------------------------

ENSTBET00000016839.1 ------------------------------------------------------------

ENSNGAT00000007851.1 ------------------------------------------------------------

ENSMUGT00000023874.1 ------------------------------------------------------------

ENSRNOT00000001611.5 ------------------------------------------------------------

MGP_CAROLIEiJ_T0023625.1 ------------------------------------------------------------

ENSMUST00000128241.8 ------------------------------------------------------------

ENSMSIT00000039370.1 ------------------------------------------------------------

MGP_SPRETEiJ_T0024771.1 ------------------------------------------------------------

ENSMAUT00000029410.1 ------------------------------------------------------------

ENSCGRT00001027434.1 ------------------------------------------------------------

ENSPEMT00000030353.2 ------------------------------------------------------------

ENSMOCT00000010482.1 ------------------------------------------------------------

ENSCCNT00000026806.1 ------------------------------------------------------------

ENSJJAT00000021205.1 CCCAGGCAAGCACATTAACCGCTCAGCCATCTCTCCAATCCTCCCCCCCAAATATTTTTA

ENSHGLT00000023656.1 ------------------------------------------------------------

ENSCLAT00000006778.1 ------------------------------------------------------------

ENSFDAT00000003362.1 ------------------------------------------------------------

ENSSVLT00005034202.1 ------------------------------------------------------------

ENSSTOT00000021109.2 ------------------------------------------------------------

ENSMMMT00000015955.1 ------------------------------------------------------------

ENSUPAT00010004184.1 ------------------------------------------------------------

ENSTBET00000016839.1 --------CTTCTGACTACAGTGTAGATGAGTACCCAGAGGGGAGCTCTGCAGCTTCAGG

ENSNGAT00000007851.1 -------CTTCCCCCAGCGAGCAGGTCTGGGTGCTCAGAGGAAA-CTCCGCAGTCC-CAA

ENSMUGT00000023874.1 -------CTTCCACCGATGCTCAGGTCTGAGCACTCAGACGTGA-CTCTGCGGCTC----

ENSRNOT00000001611.5 -------CTTCCTCCAATGCTCAGGTCTGAGCGCTCAAAGGTGA-CTC--CAGCTC-TGG

MGP_CAROLIEiJ_T0023625.1 -------CCTCCTCCAATGCTCAGGTCTGAGGGCTCAGAGGTGA-CTCTGTGACCC-TGG

ENSMUST00000128241.8 -------CCTCCTCCAATGCTCAGGTCTGAGGGCTCAGAGGTGA-CTCTGTGACCC-TGG

ENSMSIT00000039370.1 -------CCTCCTCCAATGCTCAGGTCTGAGGGCTCAGAGGTGA-CTCTCTGACCC-TGG

MGP_SPRETEiJ_T0024771.1 -------CCTCCTCCAATGCTCAGGTCTGAGGGCTCAGAGGTGA-CTCTCTGACCC-TGG

ENSMAUT00000029410.1 -------CCTCCACC--------------AGGGCTCAGAGGTGA-CTG--CGGCTC-TAT

ENSCGRT00001027434.1 -------CCTCCACC--------------AGTGCTCAGAGGTGA-CTA--CGGCTC-TAG

ENSPEMT00000030353.2 -------CCTCCTCC--------------AGCGCTCAGAGGTGA-CTG--TGGTTCGTAG

ENSMOCT00000010482.1 -------CCTCCTCC--------------AGTGCTCAGAGGTGA---CTGCGGCTC-TAG

ENSCCNT00000026806.1 ---------GCCTGTCCAGTGTGCCTTTTGATCCCCAGAGGGGC-CTCTGCAACCA-GGT

ENSJJAT00000021205.1 TTTAGTCCCTTCTTATTAAAAAGGAAATCAGAGCTGGAGGGATGGCTTAGCGGTTA-AGG

ENSHGLT00000023656.1 -----------------------GGCCTCAGCATCGAGGGGTGT-------------TGG

ENSCLAT00000006778.1 -------------------------------------AGGGAGA-------------GGC

ENSFDAT00000003362.1 ------------------------------GGGCCCAGCTGGGG----------------

ENSSVLT00005034202.1 -----------------------CCTCTGAGTAACCAAAGTGGC-CTCTGCAGCCT-AGG

ENSSTOT00000021109.2 ------------------------------------------------------------

ENSMMMT00000015955.1 -----------------------GCTCGCAGTACCCAGAGTGGC-CTCTGCAGCCT-GGA

ENSUPAT00010004184.1 -----------------------GCTCTCAGTACCCAGAGGC---CTCTGCAGCCT-GGA

ENSTBET00000016839.1 CAG--AACAG--------------------------------------------------

ENSNGAT00000007851.1 CA---AGCTG--------------------------------------------------

ENSMUGT00000023874.1 -T---GGCCC--------------------------------------------------

ENSRNOT00000001611.5 CA---GGCTG--------------------------------------------------

MGP_CAROLIEiJ_T0023625.1 CA---GGCTG--------------------------------------------------

ENSMUST00000128241.8 CA---GGCTG--------------------------------------------------

ENSMSIT00000039370.1 CA---GGCTG--------------------------------------------------

MGP_SPRETEiJ_T0024771.1 CA---GGCTG--------------------------------------------------

ENSMAUT00000029410.1 CA---GGCTG--------------------------------------------------

ENSCGRT00001027434.1 CA---GGCTG--------------------------------------------------

ENSPEMT00000030353.2 CA---GAGTG--------------------------------------------------

ENSMOCT00000010482.1 CA---GGCTG--------------------------------------------------

ENSCCNT00000026806.1 TA---GGCTG--------------------------------------------------

ENSJJAT00000021205.1 CATTTGCCTGCAAAGCCAAAGGACCCAGGTTCAATTGCCCAGGACCCATATTAGCCAGCT

ENSHGLT00000023656.1 GG---GGCTG--------------------------------------------------

ENSCLAT00000006778.1 CAGGTGCCTG--------------------------------------------------

ENSFDAT00000003362.1 -----GCCTA--------------------------------------------------

ENSSVLT00005034202.1 CA---GGCTG--------------------------------------------------

ENSSTOT00000021109.2 ------------------------------------------------------------

ENSMMMT00000015955.1 CA---GGCAG--------------------------------------------------

ENSUPAT00010004184.1 CA---GGCTG--------------------------------------------------

ENSTBET00000016839.1 ------------------------------------------------------------

ENSNGAT00000007851.1 ------------------------------------------------------------

ENSMUGT00000023874.1 ------------------------------------------------------------

ENSRNOT00000001611.5 ------------------------------------------------------------

MGP_CAROLIEiJ_T0023625.1 ------------------------------------------------------------

ENSMUST00000128241.8 ------------------------------------------------------------

ENSMSIT00000039370.1 ------------------------------------------------------------

MGP_SPRETEiJ_T0024771.1 ------------------------------------------------------------

ENSMAUT00000029410.1 ------------------------------------------------------------

ENSCGRT00001027434.1 ------------------------------------------------------------

ENSPEMT00000030353.2 ------------------------------------------------------------

ENSMOCT00000010482.1 ------------------------------------------------------------

ENSCCNT00000026806.1 ------------------------------------------------------------

ENSJJAT00000021205.1 GCACATGCGTCTGGAGTTCGTCTGCAGTGGCTGGAGGCCCTGGCACACCTATTCTCTCTC

ENSHGLT00000023656.1 ------------------------------------------------------------

ENSCLAT00000006778.1 ------------------------------------------------------------

ENSFDAT00000003362.1 ------------------------------------------------------------

ENSSVLT00005034202.1 ------------------------------------------------------------

ENSSTOT00000021109.2 ------------------------------------------------------------

ENSMMMT00000015955.1 ------------------------------------------------------------

ENSUPAT00010004184.1 ------------------------------------------------------------

ENSTBET00000016839.1 ------------------------------------------------------------

ENSNGAT00000007851.1 ------------------------------------------------------------

ENSMUGT00000023874.1 ------------------------------------------------------------

ENSRNOT00000001611.5 ------------------------------------------------------------

MGP_CAROLIEiJ_T0023625.1 ------------------------------------------------------------

ENSMUST00000128241.8 ------------------------------------------------------------

ENSMSIT00000039370.1 ------------------------------------------------------------

MGP_SPRETEiJ_T0024771.1 ------------------------------------------------------------

ENSMAUT00000029410.1 ------------------------------------------------------------

ENSCGRT00001027434.1 ------------------------------------------------------------

ENSPEMT00000030353.2 ------------------------------------------------------------

ENSMOCT00000010482.1 ------------------------------------------------------------

ENSCCNT00000026806.1 ------------------------------------------------------------

ENSJJAT00000021205.1 TCCCTCTTTCTCTGTCAAATCAGTAAATAAATAAATATTTTTTTAAAAGAATAAAAGAAA

ENSHGLT00000023656.1 ------------------------------------------------------------

ENSCLAT00000006778.1 ------------------------------------------------------------

ENSFDAT00000003362.1 ------------------------------------------------------------

ENSSVLT00005034202.1 ------------------------------------------------------------

ENSSTOT00000021109.2 ------------------------------------------------------------

ENSMMMT00000015955.1 ------------------------------------------------------------

ENSUPAT00010004184.1 ------------------------------------------------------------

ENSTBET00000016839.1 ------------------------------------------------------------

ENSNGAT00000007851.1 ------------------------------------------------------------

ENSMUGT00000023874.1 ------------------------------------------------------------

ENSRNOT00000001611.5 ------------------------------------------------------------

MGP_CAROLIEiJ_T0023625.1 ------------------------------------------------------------

ENSMUST00000128241.8 ------------------------------------------------------------

ENSMSIT00000039370.1 ------------------------------------------------------------

MGP_SPRETEiJ_T0024771.1 ------------------------------------------------------------

ENSMAUT00000029410.1 ------------------------------------------------------------

ENSCGRT00001027434.1 ------------------------------------------------------------

ENSPEMT00000030353.2 ------------------------------------------------------------

ENSMOCT00000010482.1 ------------------------------------------------------------

ENSCCNT00000026806.1 ------------------------------------------------------------

ENSJJAT00000021205.1 AAAGAAGGAAATTAAAGCCACCACGTGGGAGTTTAGTCTTGGGGGCTGGAAGGAGACACC

ENSHGLT00000023656.1 ------------------------------------------------------------

ENSCLAT00000006778.1 ------------------------------------------------------------

ENSFDAT00000003362.1 ------------------------------------------------------------

ENSSVLT00005034202.1 ------------------------------------------------------------

ENSSTOT00000021109.2 ------------------------------------------------------------

ENSMMMT00000015955.1 ------------------------------------------------------------

ENSUPAT00010004184.1 ------------------------------------------------------------

ENSTBET00000016839.1 -------------------------------CTGTGCCTATGTTGGTGGGACCAAAGCTT

ENSNGAT00000007851.1 ----------------------------GCCCTGGGCTTATATCAGTGGGACAAAAGCTC

ENSMUGT00000023874.1 ----------------------------TCCTTGGGGTGGTATCAGTAA-------GCTC

ENSRNOT00000001611.5 ----------------------------GCCCTGGGGTGGTGTCAGTAAGACAGAAGCTC

MGP_CAROLIEiJ_T0023625.1 ----------------------------GCCCTGGGGTGGTGTCAGTAAGACAGAAGCTT

ENSMUST00000128241.8 ----------------------------GCCCTGGGGTGGTGTCAGTAAGACAGAAGCTC

ENSMSIT00000039370.1 ----------------------------GTCCTGGGGTGGTGTCAGTAAGACAGAAGCTC

MGP_SPRETEiJ_T0024771.1 ----------------------------GTCCTGGGGTGGTGTCAGTAAGACAGAAGCTC

ENSMAUT00000029410.1 ----------------------------GCCCTGGGCAGGCATCCCTGGGACAGAAGCTC

ENSCGRT00001027434.1 ----------------------------GCCCTGGGCAGGCATCATTGGGACAGAAGCTC

ENSPEMT00000030353.2 ----------------------------GCCCTGGGCAGGCCCCAGTGA-----------

ENSMOCT00000010482.1 ----------------------------GCCCTGGGCA-GCATCAATGGGACAGAAG---

ENSCCNT00000026806.1 ----------------------------GCTCTGTATCTGT-CTGGTGGGACAGAAGCTC

ENSJJAT00000021205.1 AAGCACCTCCTGAGTGGGGAGGGTCTGAGTTCTATGCCCACTGGAATAGAAGAGAAGCTC

ENSHGLT00000023656.1 ------------------------------------------------------------

ENSCLAT00000006778.1 ----------------------------GTCCAG--------CCTGTGGGT---------

ENSFDAT00000003362.1 --------------------------------------CGCACCACAGGGA---------

ENSSVLT00005034202.1 ----------------------------CTTCTGTGCCCAGGTGGGTGG-ACAGATGCTC

ENSSTOT00000021109.2 ------------------------------------------------------------

ENSMMMT00000015955.1 ----------------------------CTTCTGTGCCCAGGCAGGTGGGACAGACACCC

ENSUPAT00010004184.1 ----------------------------CTTCTGTGCCCAGGCAGGTGGGACAGACACCC

ENSTBET00000016839.1 CCAGATATGGTAGAGCAGGGACTGGGGAGGGAGGGGTATCGGGTTCCCAGGAAAA-----

ENSNGAT00000007851.1 C--------------TGAGAGCCAG-TGGG------------------GAAGGGACCTGC

ENSMUGT00000023874.1 C--------------TGAGAGTCAT-TTGG------------------GGAGGACCC---

ENSRNOT00000001611.5 C--------------TGAGAGCCGG-TCGG------------------GGAGGGCCCAGC

MGP_CAROLIEiJ_T0023625.1 C--------------TAAGAGCCGG-TTGG------------------GGAGGGCCCAGC

ENSMUST00000128241.8 C--------------TAAGAGCCGG-TTGG------------------GGAGGGCCCAGC

ENSMSIT00000039370.1 C--------------TAAGAGCCCG-TTGG------------------GGAGGGCCCAGA

MGP_SPRETEiJ_T0024771.1 C--------------TAAGAGCCGG-TTGG------------------GGAGGGCCCAGA

ENSMAUT00000029410.1 C--------------CGAGAGCCAG-TTGG------------------AGAGGGCCCCGA

ENSCGRT00001027434.1 C--------------TGCGAGCCAG-TTGG------------------AGAGGGCCCTGA

ENSPEMT00000030353.2 ------------------GAGCCAG-TTGG------------------AAAGGGCCCTGC

ENSMOCT00000010482.1 ------------------------------------------------AGAGGGCCCTGC

ENSCCNT00000026806.1 C--------------TTGG-GTCAG-TAGG---GGAGAGGAGGGTCATTGGGAGACCTGT

ENSJJAT00000021205.1 T--------------TGAGAGCCAGAATGGGG----------------TGGAGGACCAGC

ENSHGLT00000023656.1 ------------------------------------------------------------

ENSCLAT00000006778.1 ---------------GAAGCATCTG-TGGGGAGCGGTGTTGGCCCCAGAGTGAGGGCAG-

ENSFDAT00000003362.1 ------------------------------------------------GGGTGGGGGCAT

ENSSVLT00005034202.1 T----------------GGAGCCTG-TAGGGAGGGGTGTGGGACTCCCAAGGGAGACTGT

ENSSTOT00000021109.2 ------------------------------------------------AGGGGAGACTGT

ENSMMMT00000015955.1 T----------------GGAGCCAG-AAAGGGAGGGGGTGGGACTCCTAGGGGAGACTGT

ENSUPAT00010004184.1 T----------------GGAGCCAGAAAGGGAGGGGGGTGGGACTCCTAGGGGAGACTGT

ENSTBET00000016839.1 GATCCTGGCTGGTGCCCCTGCCAAG---TAC

ENSNGAT00000007851.1 AACCTTGGCCAGCCCCTTACCGTGA--GTAC

ENSMUGT00000023874.1 ----------AGCTCCTCTCCCTGA--GTAC

ENSRNOT00000001611.5 AACCCTGGGTAGTTCCTCT---TGA--GTAC

MGP_CAROLIEiJ_T0023625.1 AACCCTGGCTAGCTCCTCT---TGAGTGTAC

ENSMUST00000128241.8 AACCCTGGCTAGCTCCTCT---TGAGTGTAC

ENSMSIT00000039370.1 AGCCCTGGCTAGCTCCTCT---TGAGTGTAC

MGP_SPRETEiJ_T0024771.1 AACCCTGGCTAGCTCCTCT---TGAGTGTAC

ENSMAUT00000029410.1 CATCCTGGCTAGCTCCTCTCCCTGA--GTAC

ENSCGRT00001027434.1 AACCCTGGCTAGCTCCTCTCCCTGA--GTAC

ENSPEMT00000030353.2 AACCCTGGCTAGCTCCTCTCCCTGA--GTAC

ENSMOCT00000010482.1 AACCCTGGCTAGCTCCTCTCCCTGA--GTAC

ENSCCNT00000026806.1 GACTCTGGCTGGCTCCTCCCACTGA--GTAC

ENSJJAT00000021205.1 AGCCCTCGCTGCCTTCTTCC-CTGG--GTAC

ENSHGLT00000023656.1 TGACCTGGCGGGCTCCTCCTGCTGG---TAC

ENSCLAT00000006778.1 GGGCCTGGCAGGCTCCTCCTGCTGG---TAC

ENSFDAT00000003362.1 GACCCTGGCAGGCTTCTCCT-CTGG---TAC

ENSSVLT00005034202.1 GACCATGGACGGCTCCTCCCACTGG--GTAC

ENSSTOT00000021109.2 GACCATGGCG---CTCTCCCACTGG--ATAC

ENSMMMT00000015955.1 GACCACGGC---CATCTCCCACTGG--ATAC

ENSUPAT00010004184.1 GACCATGGC---CCTCTCCCACTGG--ATAC

* ***
