# Supplementary material for: Expression analysis suggests that DNMT3L is required for oocyte de novo DNA methylation only in Muridae and Cricetidae rodents
Source: Epigenetics Chromatin. 2023 Nov 4;16:43. doi: 10.1186/s13072-023-00518-2 (PMC10625200; doi:10.1186/s13072-023-00518-2)
Supplement: Supplementary file 6 — Additional file 6: Table S1. List of additional mammalian species with genome sequences in the Ensembl database and with annotated Aire gene that were used for analysis for the presence of a potential Dnmt3l oocyte promoter within the Aire intron equivalent to mouse intron 3. Table S2. List of all rodents with genome sequences in the Ensembl database and with annotated Aire gene and a tree shrew that were used for analysis for the presence of a potential Dnmt3l oocyte promoter within the Aire intron equivalent to mouse intron 3. Table S3. Results of dN/dS ratio analysis for Dnmt3 genes in rodents. Table S4. List of Dnmt3 sequences used for computing dN/dS ratios. Sequences were downloaded from the NCBI Nucleotide database. [file 13072_2023_518_MOESM6_ESM.docx]

**Additional file table 1**

List of additional mammalian species with genome sequence in Ensembl database and with annotated Aire gene

| **Common name** | **Latin name** | **Group** | **Genome assembly** | **Intron matching mouse intron 3** |
| --- | --- | --- | --- | --- |
| Angola colobus | *Colobus angolensis palliatus* | Primates | Cang.pa_1.0 | 3 |
| Black snub-nosed monkey | *Rhinopithecus bieti* | Primates | ASM169854v1 | 3 |
| Bolivian squirrel monkey | *Saimiri boliviensis boliviensis* | Primates | SaiBol1.0 | 3 |
| Bonobo | *Pan paniscus* | Primates | panpan1.1 | 4 |
| Bushbaby | *Otolemur garnettii* | Primates | OtoGar3 | 3 |
| Capuchin | *Cebus imitator* | Primates | Cebus_imitator-1.0 | 3 |
| Chimpanzee | *Pan troglodytes* | Primates | Pan_tro_3.0 | 3 |
| Coquerel’s sifaka | *Propithecus coquereli* | Primates | Pcoq_1.0 | 3 |
| Crab-eating macaque | *Macaca fascicularis* | Primates | Macaca_fascicularis_6.0 | 3 |
| Drill | *Mandrillus leucophaeus* | Primates | Mleu.le_1.0 | 2 |
| American bison | *Bison bison bison* | Laurasiatheria | Bison_UMD1.0 | 5 (?) |
| American black bear | *Ursus americanus* | Laurasiatheria | ASM334442v1 | 5 (?) |
| American mink | *Neovison vison* | Laurasiatheria | NNQGG.v01 | 4 (?) |
| Arabian camel | *Camelus dromedarius* | Laurasiatheria | CamDro2 | 5 (?) |
| Beluga whale | *Delphinapterus leucas* | Laurasiatheria | ASM228892v3 | 4 |
| Blue whale | *Balaenoptera musculus* | Laurasiatheria | mBalMus1.v2 | 4 |
| California sea lion | *Zalophus californianus* | Laurasiatheria | mZalCal1.pri | 4 |
| Canada lynx | *Lynx canadensis* | Laurasiatheria | mLynCan4_v1.p | 4 |
| Chacoan peccary | *Catagonus wagneri* | Laurasiatheria | CatWag_v2_BIUU_UCD | 4 |
| Cat | *Felis catus* | Laurasiatheria | Felis_catus_9.0 | 4 |
| Elephant | *Loxodonta africana* | Afrotheria | Loxafr3.0 | 3 |
| Hyrax | *Procavia capensis* | Afrotheria | proCap1 | 4 |
| Armadillo | *Dasypus novemcinctus* | Xenarthra | Dasnov3.0 | 3 (?) |

(?) marks species where it was challenging to determine the matching intron

**Additional file table 2**

List of all rodents with genome sequence in Ensembl database and with annotated Aire gene and a tree shrew.

| **Common name** | **Latin name** | **Genome assembly** | **Intron matching mouse intron 3** |
| --- | --- | --- | --- |
| Algerian mouse | *Mus spretus* | SPRET_EiJ_v1 | 3 |
| Alpine marmot | *Marmota marmota marmota* | marMar2.1 | 3 |
| American beaver | *Castor canadensis* | C.can_genome_v1.0 | 3 |
| Arctic ground squirrel | *Urocitellus parryii* | ASM342692v1 | 3 |
| Chinese hamster | *Cricetulus griseus* | CriGri_1.0 | 3 |
| Damara mole-rat | *Fukomys damarensis* | DMR_v1.0 | 3 |
| Thirteen-lined ground squirrel | *Ictidomys tridecemlineatus* | SpeTri2.0 | 3 |
| Eurasian red squirrel | *Sciurus vulgaris* | mSciVul1.1 | 4 |
| Golden hamster | *Mesocricetus auratus* | MesAur1.0 | 3 |
| Lesser Egyptian jerboa | *Jaculus jaculus* | JacJac1.0 | 3 |
| Long-tailed chinchilla | *Chinchilla lanigera* | ChiLan1.0 | 3 |
| Mongolian gerbil | *Meriones unguiculatus* | MunDraft-v1.0 | 3 |
| Mouse | *Mus musculus* | GRCm39 | 3 |
| Naked mole-rat | *Heterocephalus glaber* | HetGla_female_1.0 | 3 |
| Northern American deer mouse | *Peromyscus manuculatus bairdii* | HU_Pman_2.1 | 3 |
| Prairie vole | *Microtus ochrogaster* | MicOch1.0 | 3 |
| Rat | *Rattus norvegicus* | mRatBN7.2 | 3 |
| Ryukyu mouse | *Mus caroli* | CAROLI_EIJ_v1.1 | 3 |
| Steppe mouse | *Mus spicilegus* | MUSP714 | 3 |
| Tree shrew | *Tupaia belangeri* | tupBel1 | 6 |
| Blind mole rat | *Nannospalax galili* | S.galili_v1.0 | 3 |

**Additional file table 3**

Results of dN/dS ratio analysis for Dnmt3 genes in rodents

| **Dnmt3a_model** | lnL | Free  parameters | dN/dS_0 | dN/dS  foreground |
| --- | --- | --- | --- | --- |
| M0 | -25523.05833 | 34 | 0.28034 | n/a |
| Foreground Hystricognathi | -25515.59358 | 36 | 0.257856 | 0.348231 |
| Foreground molerats | -25504.95515 | 36 | 0.25388 | 0.436515 |
|  |  |  |  |  |
|  | df | 2deltalnL | critical x2 (p = 0.95, df = 2) | critical x2 (p = 0.99 df = 2) |
| M0 vs Foreground Hystricognathi | 2 | 14.929494 | 5.991465 | 9.21034 |
| M0 vs Foreground molerats | 2 | 36.20635 | 5.991465 | 9.21034 |
|  |  |  |  |  |
|  |  |  |  |  |
| **Dnmt3b_model** | lnL | Free  parameters | dN/dS_0 | dN/dS_foreground |
| M0 | -29903.47536 | 34 | 0.33363 | n/a |
| Foreground_Hystricognathi | -29900.26129 | 36 | 0.345179 | 0.281054 |
| Foreground_molerats | -29903.32382 | 36 | 0.33503 | 0.312846 |
|  |  |  |  |  |
|  | df | 2deltalnL | critical x2 (p = 0.95, df = 2) | critical x2 (p = 0.99 df = 2) |
| M0 vs Foreground_Hystricognathi | 2 | 6.428152 | 5.991465 | 9.21034 |
| M0 vs Foreground_molerats | 2 | 0.303088 | 5.991465 | 9.21034 |
|  |  |  |  |  |
|  |  |  |  |  |
| **Dnmt3l_model** | lnL | Free  parameters | dN/dS_0 | dN/dS_foreground |
| M0 | -9278.672075 | 34 | 0.2661 | n/a |
| Foreground_Hystricognathi | -9278.671019 | 36 | 0.265732 | 0.267283 |
| Foreground_molerats | -9277.576651 | 36 | 0.259365 | 0.341208 |
|  |  |  |  |  |
|  | df | 2deltalnL | critical x2 (p = 0.95, df = 2) | critical x2 (p = 0.99 df = 2) |
| M0 vs Foreground_Hystricognathi | 2 | 0.002112 | 5.991465 | 9.21034 |
| M0 vs Foreground_molerats | 2 | 2.190848 | 5.991465 | 9.21034 |

**Additional file table 4**

List of *Dnmt3* sequences included in preparing multiple sequence alignments and computing dN/dS ratios. Sequences were downloaded from the NCBI Nucleotide database.

| **Common name** | **Latin name** | **Accession number** | | |
| --- | --- | --- | --- | --- |
|  |  | ***Dnmt3a*** | ***Dnmt3b*** | ***Dnmt3l*** |
| Alpine marmot | *Marmota marmota marmota* | XM_015479912.2 | XM_048811113.1 | XM_048803950.1 |
| Arctic ground squirrel | *Urocitellus parryii* | XM_026415245.1 | XM_026402077.1 | XM_026410108.1 |
| Common degu | *Octodon degus* | XM_023711114.1 | XM_004630806.2 | XM_023716073.1 |
| Damara mole-rat | *Fukomys damarensis* | XM_010604853.3 | XM_010628429.3 | XM_010636463.3 |
| Thirteen-lined ground squirrel | *Ictidomys tridecemlineatus* | XM_040271405.1 | XM_040275205.1 | XM_005323574.3 |
| Grey squirrel | *Sciurus carolinensis* | XM_047522091.1 | XM_047539742.1 | XM_047563246.1 |
| Guinea pig | *Cavia porcellus* | XM_013155785.2 | XM_013159580.2 | XM_003463905.2 |
| Golden hamster | *Mesocricetus auratus* | XM_040731028.1 | XM_040746525.1 | XM_040732429.1 |
| Lesser Egyptian jerboa | *Jaculus jaculus* | XM_045149784.1 | XM_045157366.1 | XM_045149220.1 |
| Long-tailed chinchilla | *Chinchilla lanigera* | XM_005400401.2 | XM_013514151.1 | XM_013506088.1 |
| Mongolian gerbil | *Meriones unguiculatus* | XM_021648279.1 | XM_021639715.1 | XM_021649724.1 |
| Mouse | *Mus musculus* | NM_001271753.2 | NM_001417024.1 | NM_001081695.2 |
| Naked mole-rat | *Heterocephalus glaber* | XM_021253571.1 | XM_021246874.1 | XM_021256378.1 |
| Rat | *Rattus norvegicus* | NM_001003958.1 | NM_001396349.1 | NM_001003964.2 |
| Reed vole | *Microtus fortis* | XM_050125868.1 | XM_050151051.1 | XM_050139877.1 |
| Tree shrew | *Tupaia belangeri* | XM_027765816.1 | XM_027774282.1 | XM_027768663.1 |
